# Comparing the accuracy of the new-generation intraocular lens power calculation formulae in axial myopic eyes: a meta-analysis

**DOI:** 10.1007/s10792-022-02466-4

**Published:** 2022-09-05

**Authors:** Hongyu Li, Zi Ye, Yu Luo, Zhaohui Li

**Affiliations:** 1 Medical School of Chinese PLA, No. 28 Fuxing Road, Haidian District, Beijing, China; 2 Senior Department of Ophthalmology, The Third Medical Center of PLA General Hospital, No. 69 Yongding Road, Haidian District, Beijing, 100080 China

**Keywords:** IOL power calculation, Cataract, Axial myopic, Meta-analysis

## Abstract

**Purpose:**

To compare the accuracy of the new-generation intraocular lens power calculation formulae in axial myopic eyes.

**Methods:**

Four databases, PubMed, Web of Science, EMBASE and Cochrane library, were searched to select relevant studies published between Apr 11, 2011, and Apr 11, 2021. Axial myopic eyes were defined as an axial length more than 24.5 mm. There are 13 formulae to participate in the final comparison (SRK/T, Hoffer Q, Holladay I, Holladay II, Haigis for traditional formulae, Barrett Universal II, Olsen, T2, VRF, EVO, Kane, Hill-RBF, LSF for the new-generation formulae). The primary outcomes were the percentage of eyes with a refractive prediction error in ± 0.5D and ± 1.0D.

**Results:**

A total of 2273 eyes in 15 studies were enrolled in the final meta-analysis. Overall, the new-generation formulae showed a relatively more accurate outcome in comparison with traditional formulae. The percentage of eyes with a predictive refraction error in ± 0.5D (± 1.0D) of Kane, EVO and LSF was higher than 80% (95%), which was only significantly different from Hoffer Q (all *P* < 0.05). Moreover, another two new-generation formulae, Barrett Universal II and Olsen, had higher percentages than SRK/T, Hoffer Q, Holladay I and Haigis for eyes with predictive refraction error in ± 0.5D and ± 1.0D (all *P* < 0.05). In ± 0.5D group, Hill-RBF was better than SRK/T (*P* = 0.02), and Holladay I was better than EVO (*P* = 0.03) and LSF (*P* = 0.009), and Hoffer Q had a lower percentage than EVO, Kane, Hill-RBF and LSF (*P* = 0.007, 0.004, 0.002, 0.03, respectively). Barrett Universal II was better than T2 (*P* = 0.02), and Hill-RBF was better than SRK/T (*P* = 0.009). No significant difference was found in other pairwise comparison.

**Conclusion:**

The new-generation formula is more accurate in intraocular lens power calculation for axial myopic eyes in comparison with the third- or fourth-generation formula.

**Supplementary Information:**

The online version contains supplementary material available at 10.1007/s10792-022-02466-4.

## Introduction

Due to the increasing number of refractive errors, more and more myopia patients are now facing cataract surgery with aging. Eyes with the axial length (AL) longer than 24.5 mm usually come with axial myopia, which are also defined as moderately long or long eyes [1–3]. It is challenging for the surgeon to select appropriate intraocular lens (IOL) power calculation formulae for axial myopic eyes, especially for the highly axial myopic eyes [4, 5]. Three main sources of errors in IOL power calculation of axial myopic eyes are the AL measurement error, anterior chamber depth (ACD) measurement error and effective lens position (ELP) error [6]. With the introduction of optical biometry, the accuracy of AL and ACD measurement has been significantly improved. Now, the main source of predictive refraction error of IOL power calculation in axial myopic eyes is ELP error which is determined by IOL formulae [6].

IOL formulae have been proposed more than 40 years to calculate IOL power after cataract surgery [5]. Over the past few decades, the third- or fourth-generation formulae (traditional formulae) still were the most common method used in IOL power calculation [7]. The third-generation formulae, including SRK/T, Hoffer Q, Holladay I, predict the postoperative ELP through the AL and corneal curvature. Earlier studies have shown that, in axial myopic eyes, SRK/T might be the most accurate formula for IOL power calculation [8]. However, some studies also suggested that the third-generation formula has no significant difference in calculating IOL power in axial myopic eyes [9, 10]. After that, the fourth-generation formulae, represented by Haigis and Holladay II, introduce other parameters, such as ACD, lens thickness and white-to-white distance, to estimate postoperative ELP and calculate IOL power. Several studies showed Haigis had a better outcome in calculating IOL power for axial myopic eyes than SRK/T, Holladay I, Holladay II and Hoffer Q formulae [11, 12]. However, consistent hyperopic errors have previously been reported in axial myopic eyes using traditional IOL power calculation formulae with ALs measured by A-scan, B-scan or optical biometry [13].

Recently, the fifth-generation formula has become available for commercial use and its performance showed promise in previous study [14]. The fifth-generation formula is also called the new-generation formula, including the Barrett Universal II (BUII), Olsen and the other new formulae [15]. Both BUII and Olsen are based on ray-tracing method and also have been shown to be a good consistent and have a great popularity in calculating IOL power for axial myopic eyes [16]. In addition, the other formulae based on artificial intelligence (AI) were also introduced to improve the accuracy of IOL power calculation, such as Kane, Hill Radial Basis Function Calculator (Hill-RBF), Ladas Super Formula (LSF) and Pearl DGS [15]. Moreover, Emmetropia Verifying Optical (EVO), T2, VRF and Naeser formula also showed acceptable accuracy in IOL power calculation [15]. With the increasing development of the new-generation IOL formulae, the predictive refraction error after cataract surgery has steadily decreased [15, 17]. Yet there is still considerably doubt about which formula provides the most accurate predictive refraction in axial myopic eyes. The purpose of this meta-analysis is to compare the accuracy of the new-generation formulae with traditional formulae and provide valuable clinical guidelines for choosing appropriate IOL power calculation formulae in axial myopic eyes.

## Patients and methods

### Study inclusion and exclusion criteria

Inclusion criteria for studies were: (1) eyes with AL longer than 24.5 mm; (2) uneventful phacoemulsification with in-bag IOL implantation; (3) at least two types of the following IOL power calculation formulae have been compared: SRK/T, Hoffer Q, Holladay I, Holladay II, Haigis, BUII, Olsen, T2, VRF, EVO, Kane, Hill-RBF, LSF; (4) biometry measurements were measured by optical biometers; (5) IOL constants were optimized; (6) postoperative refraction was performed at least two weeks for small incision surgery and 1-piece IOL implantation, more periods are needed for the others. Exclusion criteria for studies were: (1) eyes with AL shorter than 24.5 mm; (2) eyes had surgical complications or refractive surgery; (3) the percentages of eyes with refractive prediction error (PE) in ± 0.5D and ± 1.0D were unavailable. The other new formulae, Naeser 2, Panacea and Pearl-DGS, were eliminated due to the limited number of study.

### Literature search

Two independent authors (H.Y.L and Y.L) searched the following databases, including PubMed, Web of Science, EMBASE and Cochrane Library. The following search terms were used in PubMed: ("lenses, intraocular"[MeSH Terms] OR "intraocular lenses"[Title/Abstract] OR "lens intraocular"[Title/Abstract] OR "intraocular lens"[Title/Abstract] OR "IOL"[Title/Abstract] OR "IOLs"[Title/Abstract]) AND ("calculat*"[Title/Abstract] OR "formula*"[Title/Abstract]) AND ("myopi*"[Title/Abstract] OR "long eye"[Title/Abstract] OR "long axial length"[Title/Abstract] OR "long eyes"[Title/Abstract] OR "long AL"[Title/Abstract] OR "long ALs"[Title/Abstract]) AND ("cohort studies"[MeSH Terms] OR "case–control studies"[MeSH Terms] OR "comparative study"[Publication Type] OR "risk factors"[MeSH Terms] OR "cohort"[Text Word] OR "compared"[Text Word] OR "groups"[Text Word] OR "case control"[Text Word] OR "multivariate"[Text Word]) AND ("last 10 years"[PDat]). After excluding the duplicate, all possible studies were reviewed ignoring the main outcomes or languages. The two authors separately evaluated the titles and abstracts and performed a manual search by searching the reference list of all the eligible studies.

### Data extraction and quality assessment

The new-generation IOL power calculation formulae, including BUII, Olsen, T2, VRF, EVO, Kane, Hill-RBF and LSF, were compared with the traditional formulae including SRK/T, Hoffer Q, Holladay I, Holladay II, Haigis. PE was defined as the difference between the predictive spherical equivalent (SE) and the actually postoperative SE. The primary outcomes were the percentages of eyes with PE in ± 0.5D and ± 1.0D. The higher of the percentages, the better of the IOL power formulae. The two authors (H.Y.L and Y.L) independently extracted the data and compared the results. Discrepancies were resolved by another author (Z.Y). A modified checklist adapted from the QUADAS-2 tool was used to assess the quality of the evidence [18, 19]. Study characteristics extracted from the eligible studies were the first author, publication year, sample size, axial length, following-up period, postoperative refraction, IOL power formulae and the percentages of eyes with PE in ± 0.5D and ± 1.0D.

### Statistical analysis

Pooled estimates of the odds ratio (OR) were calculated with fixed-effects model when comparing the percentage of eyes with a PE in ± 0.5D and ± 1.0D of each formula. And the results were described in forest plots, with lines representing the estimated values of different studies and their confidence intervals, and the boxes graphically representing the weight assigned to each study in calculating the combined estimator of a given outcome. Substantial heterogeneity, caused by the differences across studies rather than sampling error, was defined as *I*^2^ value greater than 50%, and the P value for heterogeneity was less than 0.10. The random-effect model was used when heterogeneity was found. Sensitivity analysis and subgroup analysis were used to assess the change in overall effect when the I^2^ value was greater than 50%. Funnel plots were used to evaluate publication bias and small-study effect. Review Manager was used to data pooling (version 5.3, Cochrane Collaboration, Oxford, UK). A possibility less than 0.05 was considered to be statistically significant.

## Results

**Fig. 1:**
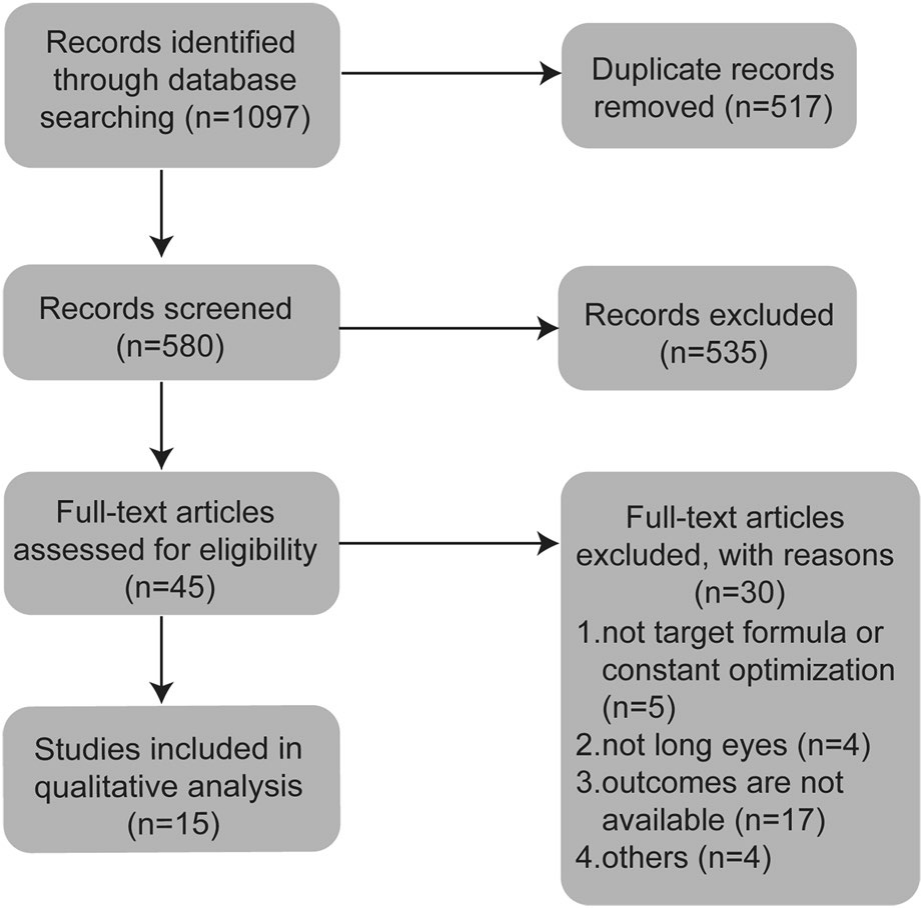
Flow diagram of articles selection

Initially, 1097 articles were identified through literature search (Fig. 1). After deleting the duplicates, 580 records remained, of which 535 records were removed because of irrelevance, such as not a clinical study, not patients without eye surgery, not comparing the accuracy of IOL formulae. Finally, 45 articles were selected for full-text assessment. Among these, five studies did not include the target formula or constant optimization, and the primary outcomes were not available in seventeen studies, and four studies evaluated eyes with axial length less than 24.5 mm, and four studies were excluded due to the other reasons, such as reviews, conference abstracts or not being an English literature. After excluding of these studies, 15 articles were used for qualitative analysis [3–5, 20–31].

### Study characteristics and quality assessment

**Fig. 2:**
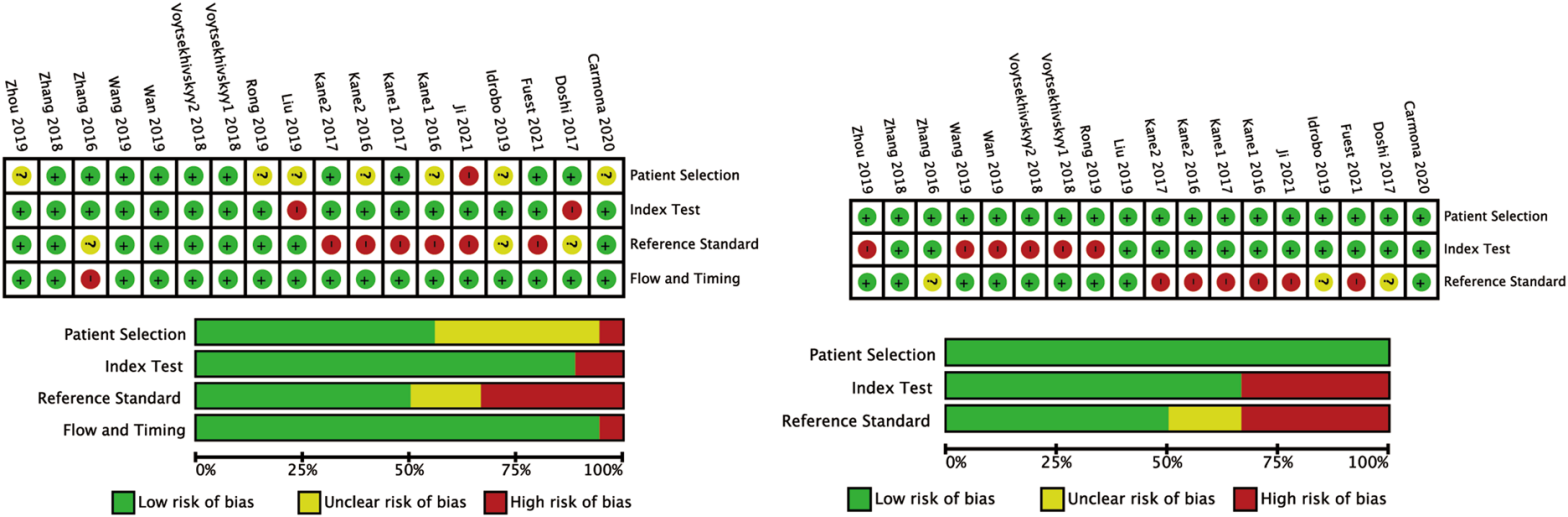
Quality assessment of the eligible studies according to the modified QUADAS-2

A total of 2273 eyes were enrolled in 15 studies (Table 1). All eligible studies included eyes after phacoemulsification with a mono-focal IOL implanting in the capsular bag. The modified QUADAS-2 was used to quality assessment (Fig. 2). Detailed information on the comprehensive assessment is described in Appendix 1. For patient selection, only one study had inappropriate exclusions, resulting in a high risk of bias [4]. Seven studies did not clarify patient enrollment methods, resulting in an unclear risk of bias. For reference standard and flow and timing assessment, six studies performed subjective refraction and three studies did not describe the refraction method. For the index test, only two studies did not declare the definition of outcomes.

**Table 1:** Characteristics of study participants

Author/year	Eyes	Male	Country	Mean age SD/range	Mean AL SD/range	Follow-up (days)	Refraction method	SRK/T	HQ	H1	H2	Haigis	BUII	Olsen	T2	VRF	EVO	Kane	RBF	LSF
Kane 2016 [3]	372	NA	Australia	NA	NA	> 14	Subjective	√	√	√	√	√	√		√					
Kane 2016 [3]	77	NA	Australia	NA	NA	> 14	Subjective	√	√	√	√	√	√		√					
Zhang 2016 [5]	171	87	China	57.65 ± 12.53	29.14 ± 2.50	> 30	NA	√	√	√		√	√							
Doshi 2017 [21]	40	25	India	59.23 ± 11.82	24.93 ± 0.80	> 28	NA	√	√	√		√								
Kane 2017 [24]	340	NA	Australia	NA	24.5–26.0	> 14	Subjective			√			√						√	
Kane 2017 [24]	47	NA	Australia	NA	NA	> 14	Subjective			√			√						√	
Voytsekhivskyy 2018 [31]	70	NA	Ukraine	NA	24.5–26.0	90	Objective	√	√	√	√	√			√	√				
Voytsekhivskyy 2018 [31]	51	NA	Ukraine	NA	NA	90	Objective	√	√	√	√	√			√	√				
Zhang 2018 [28]	63	30	China	64.0 ± 7.4	31.26 ± 1.67	30	Objective	√				√								
Idrobo 2019 [29]	63	NA	Colombia	NA	26.94 ± 1.11	> 30	NA	√		√					√					
Liu 2019 [23]	136	NA	China	61 ± 11	28.85 ± 2.02	30–90	Objective	√		√		√	√						√	
Rong 2019 [22]	79	30	China	NA	29.3 ± 2.0	30	Objective					√	√	√						
Wan 2019 [30]	127	NA	China	65.8 ± 9.1	27.72 ± 1.59	90	Objective	√	√	√		√	√						√	
Wang 2019 [20]	310	NA	America	NA	NA	> 21	Objective	√	√	√		√	√	√						
Zhou 2019 [26]	98	37	China	65.23 ± 6.78	29.63 ± 2.35	> 30	Objective	√	√	√		√	√							
Carmona 2020 [27]	115	NA	Spain	NA	NA	90	Subjective	√	√	√	√	√	√	√			√	√	√	√
Fuest 2021 [25]	58	37	Germany	61.5 ± 11.4	30.18 ± 2.67	> 28	Subjective	√		√		√	√						√	
Ji 2021 [4]	56	NA	China	62 ± 9	29.11 ± 1.98	28	Subjective	√		√	√	√	√						√	

*AL* axial length, *HQ* Hoffer Q, *H1* Holladay I, *H2* Holladay II, *BUII* Barrett Universal II, *RBF* Hill-RBF

### Outcomes

**Fig. 3:**
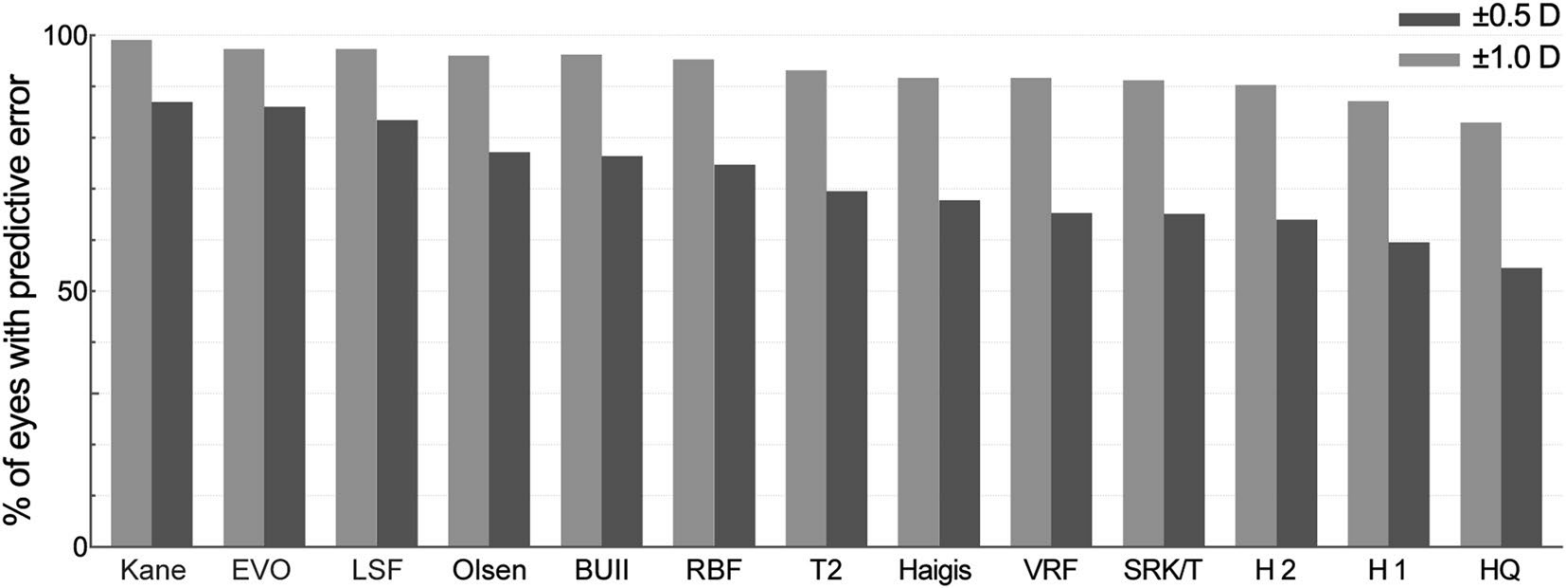
The overall percentage of refractive prediction error within ± 0.5 D and ± 1.0 D of the included formulae (HQ: Hoffer Q; H1: Holladay I; H2: Holladay II; BUII: Barrett Universal II; D: diopter)

Among the 2273 eyes included, 115 eyes were calculated with Kane, 115 with EVO, 115 with LSF, 504 with Olsen, 1986 with BUII, 879 with Hill-RBF, 633 with T2, 1823 with Haigis, 121 with VRF, 1807 with SRK/T, 741 with Holladay II, 2131 with Holladay I and 1431 with Hoffer Q. The overall percentages of eyes with PE in ± 0.5D (± 1.0D) of the above formulae are 86.96% (99.13%), 86.09% (97.39%), 83.48% (97.39%), 77.18% (96.03%), 76.38% (96.27%), 74.74% (95.34%), 69.51% (93.21%), 67.80% (91.72%), 65.29% (91.74%), 65.08% (91.20%), 63.97% (90.28%), 59.50% (87.19%) and 54.51% (83.02%), respectively (Fig. 3).

### Percentage of eyes with a PE in ± 0.5D

**Fig. 4:**
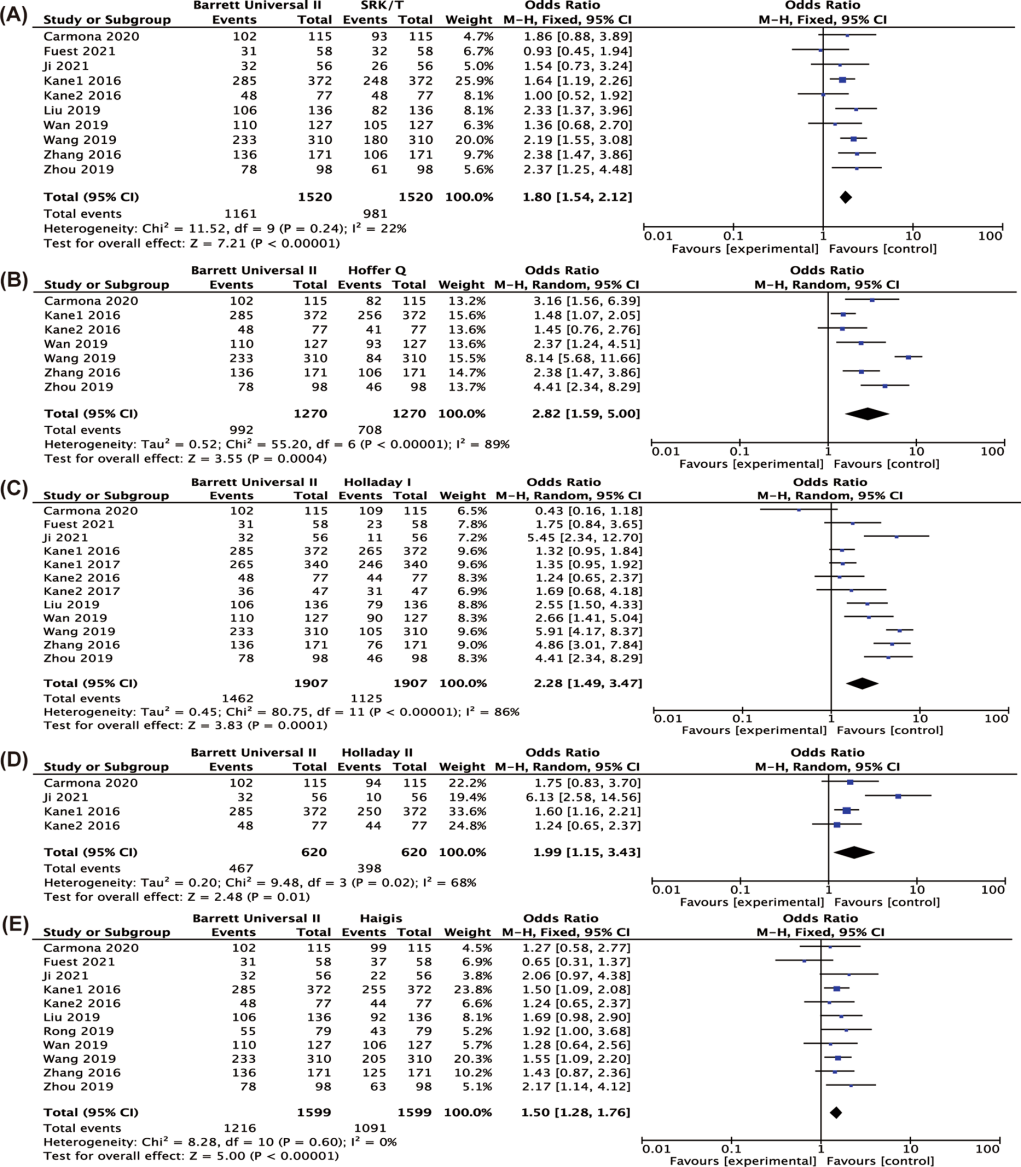

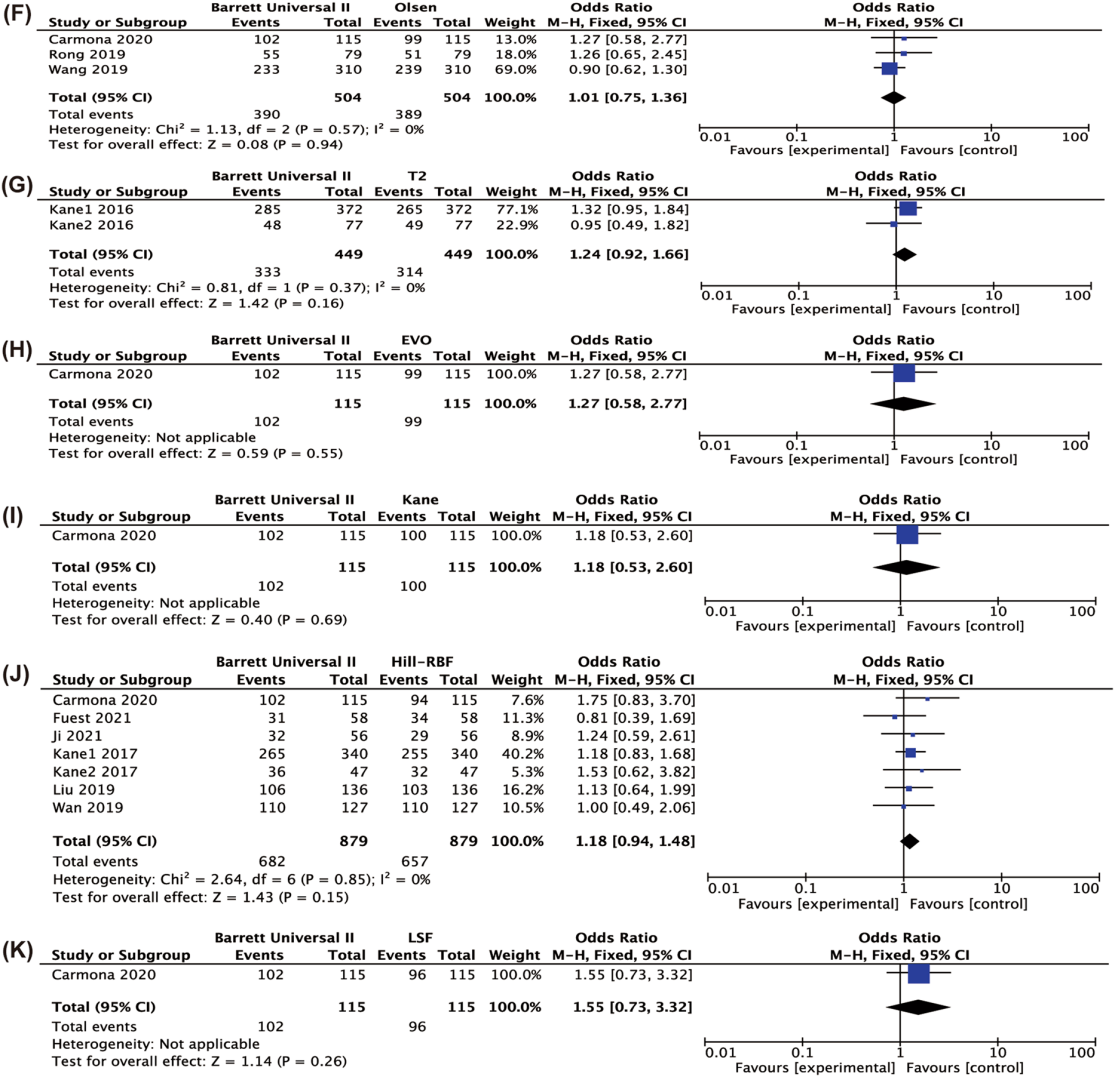
Forest plots of the percentage of eyes with refractive prediction error in ± 0.5 D when comparing Barrett Universal II with SRK/T (**A**), Hoffer Q (**B**), Holladay I (**C**), Holladay II (**D**), Haigis (**E**), Olsen (**F**), T2 (**G**), EVO (**H**), Kane (**I**), H-RBF (**J**), LSF (**K**)

Figure 4 shows the difference in the percentage of eyes with a PE in ± 0.5D comparing BUII with the other formulae. BUII was more accurate than SRK/T (*P* < 0.001), Hoffer Q (*P* < 0.001), Holladay I (*P* < 0.001), Holladay II (*P* = 0.01) and Haigis (*P* < 0.001). Appendices 2–7 show the results of pairwise comparisons of other formulae in terms of the percentages of eyes with PE in ± 0.5D. SRK/T had a significantly higher percentage of eyes with PE in ± 0.5D than Hoffer Q (*P* = 0.009) and lower of that than Haigis, Olsen and Hill-RBF (Appendix 2, *P* = 0.02, < 0.001, = 0.02, respectively). And Hoffer Q also had the lowest percentage when comparing with Haigis, Olsen, EVO, Kane, Hill-RBF and LSF (Appendix 3, *P* = 0.02, 0.01, 0.007, 0.004, 0.002, 0.03, respectively). Additionally, significant differences were found between Holladay I and Holladay II, EVO and LSF (Appendix 4, *P* = 0.02, 0.03 and 0.009, respectively) and between Olsen and Haigis (Appendix 6, *P* = 0.003).

### Percentage of eyes with a PE in ± 1.0D

**Fig. 5:**
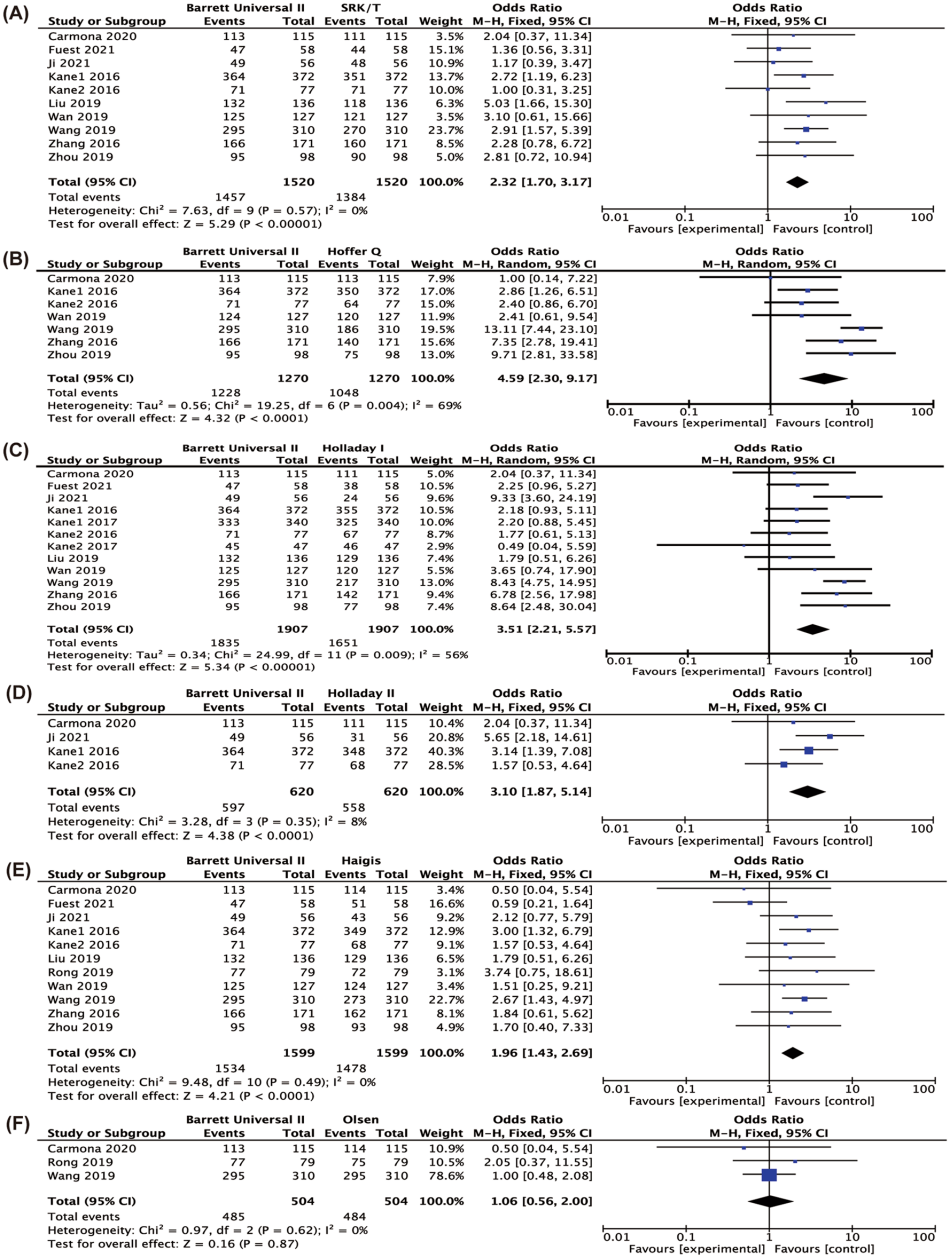

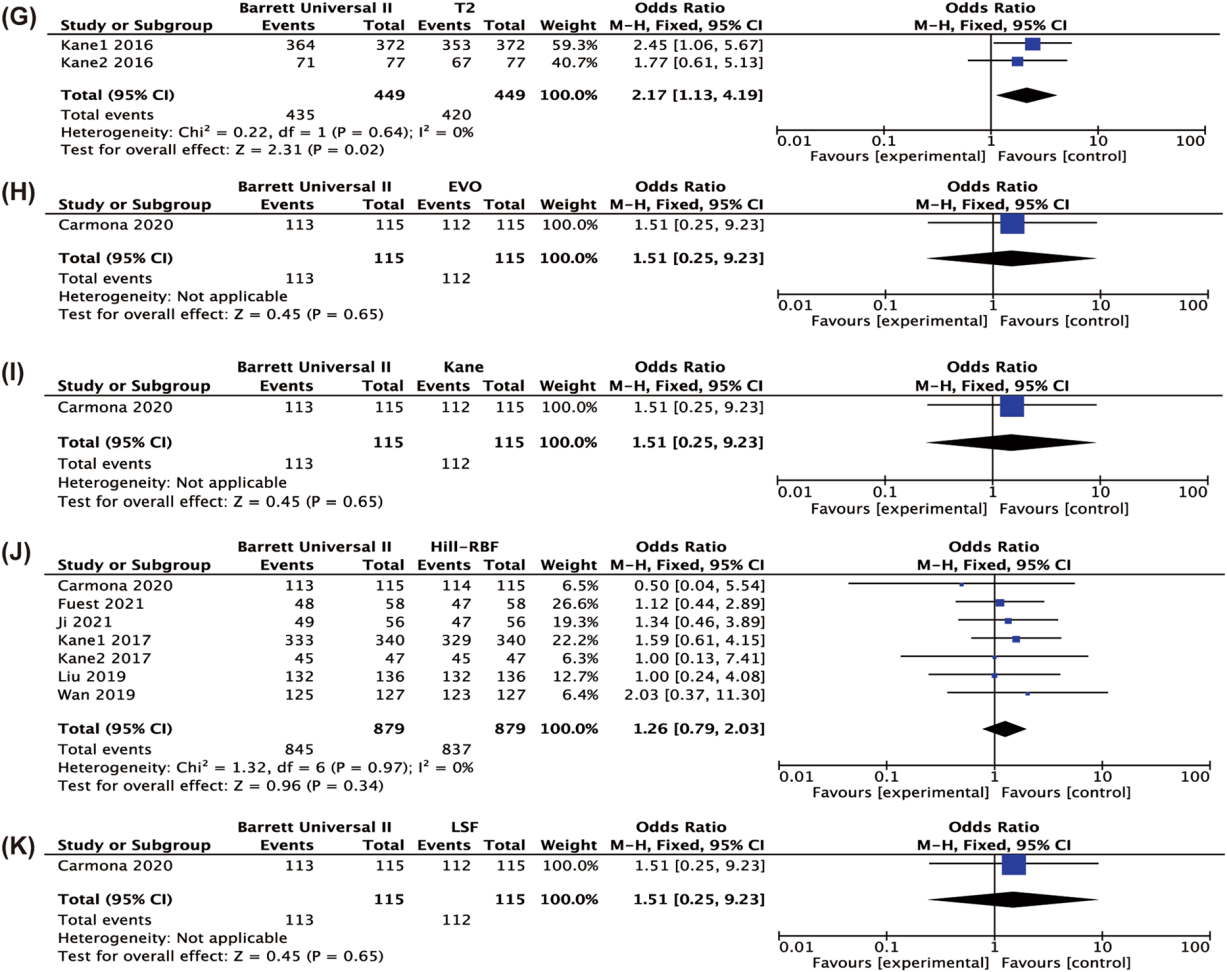
Forest plots of the percentage of eyes with refractive prediction error in ± 1.0 D when comparing Barrett Universal II with SRK/T (**A**), Hoffer Q (**B**), Holladay I (**C**), Holladay II (**D**), Haigis (**E**), Olsen (**F**), T2 (**G**), EVO (**H**), Kane (**I**), H-RBF (**J**), LSF (**K**)

The comparison between BUII with the others in terms of percentage of eyes with a PE in ± 1.0D is shown in Fig. 5. The percentage of eyes with a PE in ± 1.0D calculated by the BUII formula was significantly higher than the SRK/T (*P* < 0.001), Hoffer Q (*P* < 0.001), Holladay I (*P* < 0.001), Holladay II (*P* < 0.001), Haigis (*P* < 0.001) and T2 (*P* = 0.02). Appendices 8–13 show the results of pairwise comparisons of other formulae in terms of the percentages of eyes with PE in ± 1.0D. SRK/T produced a higher percentage than Hoffer Q (*P* = 0.006) and Haigis (*P* = 0.03), but a lower of that than Olsen and Hill-RBF (Appendix 8, *P* = 0.003 and 0.009, respectively). Additionally, Hoffer Q was less accurate than Holladay I, Haigis and Olsen (Appendix 9, *P* = 0.007, 0.03, 0.02, respectively). And a higher percentage of eyes with PE in ± 1.0D was also found in Olsen when comparing with Holladay I (Appendix 10, *P* < 0.001) and Haigis (Appendix 12, *P* = 0.002).

### Heterogeneity and subgroup analysis

Forest plots showed the *I*^2^ values, mean difference and 95% confidence interval (CI). Substantial heterogeneity was detected in 11 pairs, and then the random-effect model was used (Appendix 14–15). Sensitivity analysis indicated that I^2^ value decreased to 0% in the comparison of the percentage of eyes with PE in ± 0.5D between BUII and Holladay II by omitting Ji 2021 (Appendix 14C, *P* < 0.001). In addition, substantial heterogeneity was decreased to insignificant when the enrolled eyes were sub-grouped by the biometry measurement difference (IOL Master and Lenstar). The results showed that there were significant differences in comparison of the percentage of eyes with PE in ± 0.5D (1.0D) between BUII and Hoffer Q and Holladay I in both subgroups (Appendix 14 A-B and 15 A-B, all *P* < 0.01). Similar results were also found when comparing SRK/T with Hoffer Q and Holladay I in ± 0.5D group and Haigis with SRK/T and Hoffer Q in ± 1.0D group (all *P* < 0.05). Funnel plot is shown in Appendix 16.

## Discussion

The widespread application of phacoemulsification combined with in-bag IOL implantation has led to the improvement of surgical techniques and the reduction of surgical complications. Therefore, the postoperative refractive status is less affected by surgical factors than it was in the past. Yet the utmost importance that affects the refractive status after cataract surgery is the accuracy of IOL power calculation which is closely related to the choice of IOL formula. A previous meta-analysis by Wang et al. compared the accuracy of different IOL formulae in axial myopic eyes and demonstrated the superiority of BUII over SRK/T, Hoffer Q, Holladay I and Holladay II [32]. However, they did not compare the newest formulae based on ray tracing or AI methods.

Previous studies indicated that several measures could be used to evaluate the accuracy of IOL formulae, such as MAE, median absolute prediction error (MedAE), as well as the percentage of eyes with PE in ± 0.5D and ± 1.0D and > 2.00D [33]. It is better to compare MedAE and MAE when comparing the accuracy of different IOL power calculation formulae. However, both MedAE and MAE are abnormal Gaussian distribution and it is also difficult to obtain standard deviation values, resulting in the impossibility to compare the MedAE and MAE in this meta-analysis. Therefore, we choose to compare the percentage of eyes with PE in ± 0.5D and ± 1.0D. In addition, as the central cornea alter to the flattest area after myopic ablation, the ratio between anterior and posterior corneal curvature would decrease [34]. This meta-analysis excluded myopic eyes with previous refractive surgery because of the corneal power measurement error and ELP error after laser ablation [35, 36]. Moreover, in the present study, we included studies that used the optical biometer (IOL Master or Lenstar) for biometry measurements, which was recommended as the best precision of measurements [33]. Postoperative refraction should be measured at least 2 weeks, because it could be considered to be stable at 1 week after small incision cataract surgery with a 1-piece IOL implantation [37, 38]. In order to control heterogeneity and biasness, we also excluded the studies which did not perform the constant optimization procedure. Additionally, the new-generation IOL formulae (BUII, Olsen, T2, VRF, EVO, Kane, Hill-RBF and LSF) were compared with traditional ones (SRK/T, Hoffer Q, Holladay I, Holladay II, Haigis). To our knowledge, this is the first meta-analysis to assess the accuracy of the new-generation IOL power calculation formulae in axial myopic eyes by comparing the percentage of eyes with PE in ± 0.5D and ± 1.0D.

Traditionally, IOL power calculation formulae were classified by generation. For example, SRK II is a second-generation formula; SRK/T, Hoffer Q and Holladay I are third-generation formulae; Holladay II and Haigis are fourth generation; Barrett Universal II, Olsen and another new formula are fifth generation, which also called the new generation. Over the past four decades, cataract surgeons usually applied the third- or fourth-generation formulae to calculate the IOL power in virgin eyes. Previous studies showed that, for axial myopic eyes, SRK/T was slightly better than Hoffer Q and Holladay I [39–41]. Similar results were found in this meta-analysis. However, all third-generation formulae showed a lower percentage of eyes with PE in ± 0.5D and ± 1.0D than Haigis. The disadvantage of third-generation formulae is that only two variables (AL and corneal power) are used to determine the postoperative IOL position, making it inaccurate to evaluate the actual size of the anterior segment of the eyes. The Haigis formula adds ACD and the Holladay II adds ACD, lens thick, corneal diameter, pre-refraction and gender to calculate the postoperative IOL position [42]. Although the fourth-generation formulae introduce more parameters to estimate the ELP, most traditional formulae could make a hyperopic drift postoperatively, and even the longer the axial length is, the higher the PE is made. It might be associated with a slight but significant reduction in the AL measurement after cataract surgery. Maddalena et al. pointed out two hypotheses to expound the differences in AL measurement, including the decrease in the volume of the eye postoperatively and the wrong refractive index of the lens due to the cataract grade preoperatively [43].

In order to improve the accuracy of IOL power calculation, it has recently come up with several new formulae, collectively called the fifth-generation formulae, such as BUII, Olsen and formulae based on AI [15]. Each of these formulae uses at least five variables to calculate IOL position. The BUII is the evolution of the Barrett Universal I, published as a thick-lens paraxial formula [44]. Kane et al. have proved that BUII is the most accurate formula in comparison with the third- and fourth- generation formulae for IOL power calculation in medium, medium-long and long eyes [3]. It showed a higher percentage of eyes with PE in ± 0.5D and a lower MAE as well as MedAE. In our meta-analysis, BUII also showed a higher percentage of eyes with PE in ± 0.5D and ± 1.0D than SRK/T, Hoffer Q, Holladay I, Holladay II and Haigis. In addition, our study included Olsen formula which is based on ray-tracing method and uses a special C-constant to estimate ELP [45]. C-constant is based on ACD, lens thickness and IOL constant, and no longer dependent on AL or corneal power. Meanwhile, it also considers corneal irregularity, pupil diameter and IOL thickness to minimize the aberration. Melles and Rong demonstrated its superiority for IOL power calculation in comparison with traditional formulae [16, 22]. Our meta-analysis also found the accuracy of Olsen could be comparable to BUII, which is significantly better than the third- and fourth-generation formulae.

Except for all above-mentioned formulae, there are a lot of the other new formulae being proposed to calculate IOL power, where T2 is a modification of the original SRK/T, and VRF or EVO is a vergence formula even it is unpublished, as well as several formulae based on AI, such as Kane, Hill-RBF and LSF [15, 46]. Although all these formulae were reported high accuracy, they have been tested by a few studies [30, 31]. Hill-RBF is the first AI formula installed on the Lenstar, which uses pattern recognition and data interpolation to calculate the IOL power; Kane is a new formula based on theoretical optics and contains some element of AI, but its structure is largely unknown; LSF uses the postoperative data of more than 4000 eyes to build a three-dimensional model to calculate IOL power [24, 30, 47]. It recently was reported that EVO and Hill-RBF had a more popularity and excellent outcome in IOL power calculation for axial myopic eyes [30]. In this meta-analysis, we found that Hill-RBF had a significantly higher percentage of eyes with PE in ± 0.5D and ± 1.0D than SRK/T. No statistical difference was found between the other new formulae and traditional formulae. Interestingly, our study indicated that Kane, EVO and LSF showed a higher percentage of eyes with PE in ± 0.5D and ± 1.0D than the others, even without statistically significant differences. The possible reason might be that all three formulae are created with “big data” techniques and using several basic parameters to make its predictions [48, 49]. Due to the limit number of the enrolled eyes with VRF, EVO, LSF and Kane, it is still difficult to determine which new formulae are the most accurate one for IOL power calculation in axial myopic eyes.

This meta-analysis has several limitations. Firstly, a few of the enrolled studies were retrospective settings with a small sample size, which could cause a selective bias that is relative to the variability of patient characteristics, multiple IOL types and a single-center study. However, it is considered acceptably to compare the accuracy of IOL power calculation formulae [50]. Second, although the AL of all included eyes was longer than 24.5 mm, the scope of AL was different between different studies. This would have a small effect on the eventual results. Next, one of the enrolled study might include both eyes of the same patient, which could lead smaller P-values [23]. But there were only 136 eyes of 92 patients in this study. It might not significantly affect the final results of 2273 eyes. Finally, only one study included the EVO, Kane and LSF. Therefore, the results in comparison with these three formulae have a limited application. Future studies need to investigate the accuracy of these results for IOL power calculation in axial myopic eyes.

## Conclusions

This meta-analysis reveals promising results for the new-generation formula, such as Kane, EVO, LSF, BUII and Hill-RBF, in IOL power calculation for axial myopic eyes. These new formulae have a higher percentage of eyes with a PE both in ± 0.5 D and ± 1.0 D than traditional formulae, such as SRK/T, Hoffer Q, Holladay I/II and Haigis.

## Supplementary Information

Below is the link to the electronic supplementary material.

Supplementary file1 (PDF 74 kb)

Supplementary file2 (TIF 37288 kb)

Supplementary file3 (TIF 29959 kb)

Supplementary file4 (TIF 28856 kb)

Supplementary file5 (TIF 28486 kb)

Supplementary file6 (TIF 24823 kb)

Supplementary file7 (TIF 28927 kb)

Supplementary file8 (TIF 37811 kb)

Supplementary file9 (TIF 30212 kb)

Supplementary file10 (TIF 30066 kb)

Supplementary file11 (TIF 28607 kb)

Supplementary file12 (TIF 25181 kb)

Supplementary file13 (TIF 29976 kb)

Supplementary file14 (TIF 33923 kb)

Supplementary file15 (TIF 40038 kb)

Supplementary file16 (TIF 25037 kb)

Supplementary file17 (DOCX 21 kb)

## Data Availability

The datasets supporting the conclusions of this article are included within the article and its additional file.

## References

[1] Chong EW, Mehta JS (2016). High myopia and cataract surgery. Curr Opin Ophthalmol.

[2] Olsen T, Thim K, Corydon L (1991). Accuracy of the newer generation intraocular lens power calculation formulas in long and short eyes. J Cataract Refract Surg.

[3] Kane JX, Van Heerden A, Atik A, Petsoglou C (2016). Intraocular lens power formula accuracy: comparison of 7 formulas. J Cataract Refract Surg.

[4] Ji J, Liu Y, Zhang J, Wu X, Shao W, Ma B, Luo M (2021). Comparison of six methods for the intraocular lens power calculation in high myopic eyes. Eur J Ophthalmol.

[5] Zhang Y, Liang XY, Liu S, Lee JW, Bhaskar S, Lam DS (2016). Accuracy of intraocular lens power calculation formulas for highly myopic eyes. J Ophthalmol.

[6] Hill WE, Abulafia A, Wang L, Koch DD (2017) Pursuing perfection in IOL calculations. II. Measurement foibles: measurement errors, validation criteria, IOL constants, and lane length. J Cataract Refract Surg 43(7):869–70. http://doi.org/10.1016/j.jcrs.2017.07.006 10.1016/j.jcrs.2017.07.006 28823428

[7] Fayette RM, Cakiner-Egilmez T (2015). What factors affect intraocular lens power calculation?. Insight.

[8] Aristodemou P, Knox Cartwright NE, Sparrow JM, Johnston RL (2011). Formula choice: Hoffer Q, Holladay 1, or SRK/T and refractive outcomes in 8108 eyes after cataract surgery with biometry by partial coherence interferometry. J Cataract Refract Surg.

[9] Cooke DL, Cooke TL (2016). Comparison of 9 intraocular lens power calculation formulas. J Cataract Refract Surg.

[10] Wang L, Shirayama M, Ma XJ, Kohnen T, Koch DD (2011) Optimizing intraocular lens power calculations in eyes with axial lengths above 25.0 mm. J Cataract Refract Surg 37(11):2018–2027. http://doi.org/10.1016/j.jcrs.2011.05.042 10.1016/j.jcrs.2011.05.042 22018365

[11] Bang S, Edell E, Yu Q, Pratzer K, Stark W (2011). Accuracy of intraocular lens calculations using the IOLMaster in eyes with long axial length and a comparison of various formulas. Ophthalmology.

[12] Chen C, Xu X, Miao Y, Zheng G, Sun Y, Xu X (2015). Accuracy of intraocular lens power formulas involving 148 eyes with long axial lengths: a retrospective chart-review study. J Ophthalmol.

[13] Maclaren RE, Sagoo MS, Restori M, Allan BD (2005). Biometry accuracy using zero- and negative-powered intraocular lenses. J Cataract Refract Surg.

[14] Roberts TV, Hodge C, Sutton G, Lawless M, Vision Eye Inst IOLO (2018). Comparison of Hill-radial basis function, Barrett Universal and current third generation formulas for the calculation of intraocular lens power during cataract surgery. Clin Exp Ophthalmol.

[15] Savini G, Taroni L, Hoffer KJ (2020) Recent developments in intraocular lens power calculation methods-update 2020. Ann Transl Med 8(22):1553. http://doi.org/10.21037/atm-20-2290 10.21037/atm-20-2290 PMC7729321 33313298

[16] Melles RB, Holladay JT, Chang WJ (2018). Accuracy of intraocular lens calculation formulas. Ophthalmology.

[17] Hipolito-Fernandes D, Elisa Luis M, Gil P, Maduro V, Feijao J, Yeo TK, Voytsekhivskyy O, Alves N (2020). VRF-G, a new intraocular lens power calculation formula: a 13-formulas comparison study. Clin Ophthalmol.

[18] Deshpande SN, Van Asselt AD, Tomini F, Armstrong N, Allen A, Noake C, Khan K, Severens JL, Kleijnen J, Westwood ME (2013). Rapid fetal fibronectin testing to predict preterm birth in women with symptoms of premature labour: a systematic review and cost analysis. Health Technol Assess.

[19] Wang Q, Jiang W, Lin T, Wu X, Lin H, Chen W (2018). Meta-analysis of accuracy of intraocular lens power calculation formulas in short eyes. Clin Exp Ophthalmol.

[20] Wang L, Cao D, Weikert MP, Koch DD (2019). Calculation of axial length using a single group refractive index versus using different refractive indices for each ocular segment: theoretical study and refractive outcomes. Ophthalmology.

[21] Doshi D, Limdi P, Parekh N, Gohil N (2017) A comparative study to assess the predictability of different iol power calculation formulas in eyes of short and long axial length. J Clin Diagn Res 11(1):NC01–NC4. http://doi.org/10.7860/JCDR/2017/22095.9136 10.7860/JCDR/2017/22095.9136 PMC5324431 28273986

[22] Rong X, He W, Zhu Q, Qian D, Lu Y, Zhu X (2019). Intraocular lens power calculation in eyes with extreme myopia: Comparison of Barrett Universal II, Haigis, and Olsen formulas. J Cataract Refract Surg.

[23] Liu J, Wang L, Chai F, Han Y, Qian S, Koch DD, Weikert MP (2019). Comparison of intraocular lens power calculation formulas in Chinese eyes with axial myopia. J Cataract Refract Surg.

[24] Kane JX, Van Heerden A, Atik A, Petsoglou C (2017). Accuracy of 3 new methods for intraocular lens power selection. J Cataract Refract Surg.

[25] Fuest M, Plange N, Kuerten D, Schellhase H, Mazinani BAE, Walter P, Kohnen S, Widder RA, Roessler G (2021). Intraocular lens power calculation for plus and minus lenses in high myopia using partial coherence interferometry. Int Ophthalmol.

[26] Zhou D, Sun Z, Deng G (2019). Accuracy of the refractive prediction determined by intraocular lens power calculation formulas in high myopia. Indian J Ophthalmol.

[27] Carmona-Gonzalez D, Castillo-Gomez A, Palomino-Bautista C, Romero-Dominguez M, Gutierrez-Moreno MA (2020). Comparison of the accuracy of 11 intraocular lens power calculation formulas. Eur J Ophthalmol.

[28] Zhang Z, Miao Y, Fang X, Luo Q, Wang Y (2018) Accuracy of the Haigis and SRK/T Formulas in Eyes Longer than 29.0 mm and the Influence of Central Corneal Keratometry Reading. Curr Eye Res 43(11):1316–21. http://doi.org/10.1080/02713683.2018.1488265 10.1080/02713683.2018.1488265 29958004

[29] Idrobo-Robalino CA, Santaella G, Gutierrez AM (2019) T2 formula in a highly myopic population, comparison with other methods and description of an improved approach for estimating corneal height. BMC Ophthalmol 19(1). http://doi.org/10.1186/s12886-019-1226-7 10.1186/s12886-019-1226-7 PMC6849235 31718610

[30] Wan KH, Lam TCH, Yu MCV, Chan TCY (2019). Accuracy and precision of intraocular lens calculations using the new Hill-RBF Version 2.0 in eyes with high axial myopia. Am J Ophthalmol.

[31] Voytsekhivskyy OV (2018). Development and clinical accuracy of a new intraocular lens power formula (VRF) compared to other formulas. Am J Ophthalmol.

[32] Wang Q, Jiang W, Lin T, Zhu Y, Chen C, Lin H, Chen W (2018). Accuracy of intraocular lens power calculation formulas in long eyes: a systematic review and meta-analysis. Clin Exp Ophthalmol.

[33] Hoffer KJ, Aramberri J, Haigis W, Olsen T, Savini G, Shammas HJ, Bentow S (2015). Protocols for studies of intraocular lens formula accuracy. Am J Ophthalmol.

[34] Mcalinden C (2012). Corneal refractive surgery: past to present. Clin Exp Optom.

[35] Alio JL, Abdelghany AA, Abdou AA, Maldonado MJ (2016). Cataract surgery on the previous corneal refractive surgery patient. Surv Ophthalmol.

[36] Savini G, Hoffer KJ (2018). Intraocular lens power calculation in eyes with previous corneal refractive surgery. Eye Vis.

[37] Nejima R, Miyai T, Kataoka Y, Miyata K, Honbou M, Tokunaga T, Kawana K, Kiuchi T, Oshika T (2006) Prospective intrapatient comparison of 6.0-millimeter optic single-piece and 3-piece hydrophobic acrylic foldable intraocular lenses. Ophthalmology 113(4):585–590. http://doi.org/10.1016/j.ophtha.2005.10.064 10.1016/j.ophtha.2005.10.064 16581420

[38] Lyle WA, Jin GJ (1996). Prospective evaluation of early visual and refractive effects with small clear corneal incision for cataract surgery. J Cataract Refract Surg.

[39] Holladay JT, Prager TC, Chandler TY, Musgrove KH, Lewis JW, Ruiz RS (1988). A three-part system for refining intraocular lens power calculations. J Cataract Refract Surg.

[40] Hoffer KJ (1993). The Hoffer Q formula: a comparison of theoretic and regression formulas. J Cataract Refract Surg.

[41] Retzlaff JA, Sanders DR, Kraff MC (1990). Development of the SRK/T intraocular lens implant power calculation formula. J Cataract Refract Surg.

[42] Haigis W, Lege B, Miller N, Schneider B (2000). Comparison of immersion ultrasound biometry and partial coherence interferometry for intraocular lens calculation according to Haigis. Graefes Arch Clin Exp Ophthalmol.

[43] De Bernardo M, Salerno G, Cornetta P, Rosa N (2018). Axial length shortening after cataract surgery: new approach to solve the question. Transl Vis Sci Technol.

[44] Barrett GD (1993). An improved universal theoretical formula for intraocular lens power prediction. J Cataract Refract Surg.

[45] Olsen T, Hoffmann P (2014). C constant: new concept for ray tracing-assisted intraocular lens power calculation. J Cataract Refract Surg.

[46] Sheard RM, Smith GT, Cooke DL (2010). Improving the prediction accuracy of the SRK/T formula: the T2 formula. J Cataract Refract Surg.

[47] Ladas JG, Siddiqui AA, Devgan U, Jun AS (2015). A 3-D "Super Surface" combining modern intraocular lens formulas to generate a "Super Formula" and maximize accuracy. JAMA Ophthalmol.

[48] Savini G, Hoffer KJ, Balducci N, Barboni P, Schiano-Lomoriello D (2020). Comparison of formula accuracy for intraocular lens power calculation based on measurements by a swept-source optical coherence tomography optical biometer. J Cataract Refract Surg.

[49] Connell BJ, Kane JX (2019). Comparison of the Kane formula with existing formulas for intraocular lens power selection. BMJ Open Ophthalmol.

[50] Wang L, Koch DD, Hill W, Abulafia A (2017) Pursuing perfection in intraocular lens calculations: III. Criteria for analyzing outcomes. J Cataract Refract Surg 43(8):999–1002. http://doi.org/10.1016/j.jcrs.2017.08.003 10.1016/j.jcrs.2017.08.003 28917430

