# Supplementary material for: Comparing the accuracy of the new-generation intraocular lens power calculation formulae in axial myopic eyes: a meta-analysis
Source: Int Ophthalmol. 2022 Sep 5;43(2):619–33. doi: 10.1007/s10792-022-02466-4 (PMC9971158; doi:10.1007/s10792-022-02466-4)
Supplement: Supplementary file 1 — Supplementary file1 (PDF 74 kb) [file 10792_2022_2466_MOESM1_ESM.pdf]

Appendix 1. Modified Check-list Adapted from the QUADAS-2 Tool

Assessment of Risk of Bias

Domain 1: Patient Selection

Question 1: Was a consecutive or random sample of patients enrolled?
'yes' → low risk of bias
'unclear' → unclear risk of bias
'no' → high risk of bias

Question 2: Did the study avoid inappropriate exclusions?
'no' for < 10% of patients or 'yes' → low risk of bias
'unclear' → unclear risk of bias
'no' for ≥ 10% of patients → high risk of bias

Domain 2: Index Test

Question: Were the index test result read without knowing the result of the reference standard?
'yes' → low risk of bias
'unclear' → unclear risk of bias
'no' → high risk of bias

Domain 3: Reference Standard

Question: The method in testing the postoperative refraction
'objective refraction' → low risk of bias
'unclear' → unclear risk of bias
'subjective refraction' → high risk of bias

Domain 4: Flow and Timing

Question 1: Were all patients included in the analysis?
'no' but for < 10% of patients or 'yes' → low risk of bias
'unclear' → unclear risk of bias
'no' for ≥ 10% of patients → high risk of bias

Question 2: Was there appropriate interval between the surgery and reference standard?
14 days after surgery or later → low risk of bias
'unclear' → unclear risk of bias
before 14 days postoperatively → high risk of bias

Assessment of Applicability Concerns

Domain 1: Patient Selection

Question: Was a different diagnosis presented?
'no' → low risk of bias
'unclear' → unclear risk of bias
'yes' → high risk of bias

Domain 2: Index Test

Question: Variations in test technology
'no' → low risk of bias
'unclear' → unclear risk of bias
'yes' → high risk of bias

Domain 3: Reference Standard

Question: The method in testing the postoperative refraction
'objective refraction' → low risk of bias
'unclear' → unclear risk of bias
'subjective refraction' → high risk of bias

Author/Year	Risk of Bias				Applicability Concerns		
	patient selection	index test	reference standard	flow and timing	patient selection	index test	reference standard
**Kane1 2016**	U	L	H	L	L	L	H
**Kane2 2016**	U	L	H	L	L	L	H
**Zhang 2016**	L	L	U	H	L	L	U
**Doshi 2017**	L	H	U	L	L	L	U
**Kane1 2017**	L	L	H	L	L	L	H
**Kane2 2017**	L	L	H	L	L	L	H
**Vogteskhivskyy1 2018**	L	L	L	L	L	H	L
**Vogteskhivskyy2 2018**	L	L	L	L	L	H	L
**Zhang 2018**	L	L	L	L	L	L	L
**Idrobo 2019**	U	L	U	L	L	L	U
**Liu 2019**	U	H	L	L	L	L	L
**Rong 2019**	U	L	L	L	L	H	L
**Wan 2019**	L	L	L	L	L	H	L
**Wang 2019**	L	L	L	L	L	H	L
**Zhou 2019**	U	L	L	L	L	H	L
**Carmona 2020**	U	L	L	L	L	L	L
**Fuest 2021**	L	L	H	L	L	L	H
**Ji 2021**	H	L	H	L	L	L	H

L: low risk; H: high risk; U: unclear risk
