# Supplementary figures and images for: Comparing the accuracy of the new-generation intraocular lens power calculation formulae in axial myopic eyes: a meta-analysis
Source: Int Ophthalmol. 2022 Sep 5;43(2):619–33. doi: 10.1007/s10792-022-02466-4 (PMC9971158; doi:10.1007/s10792-022-02466-4)

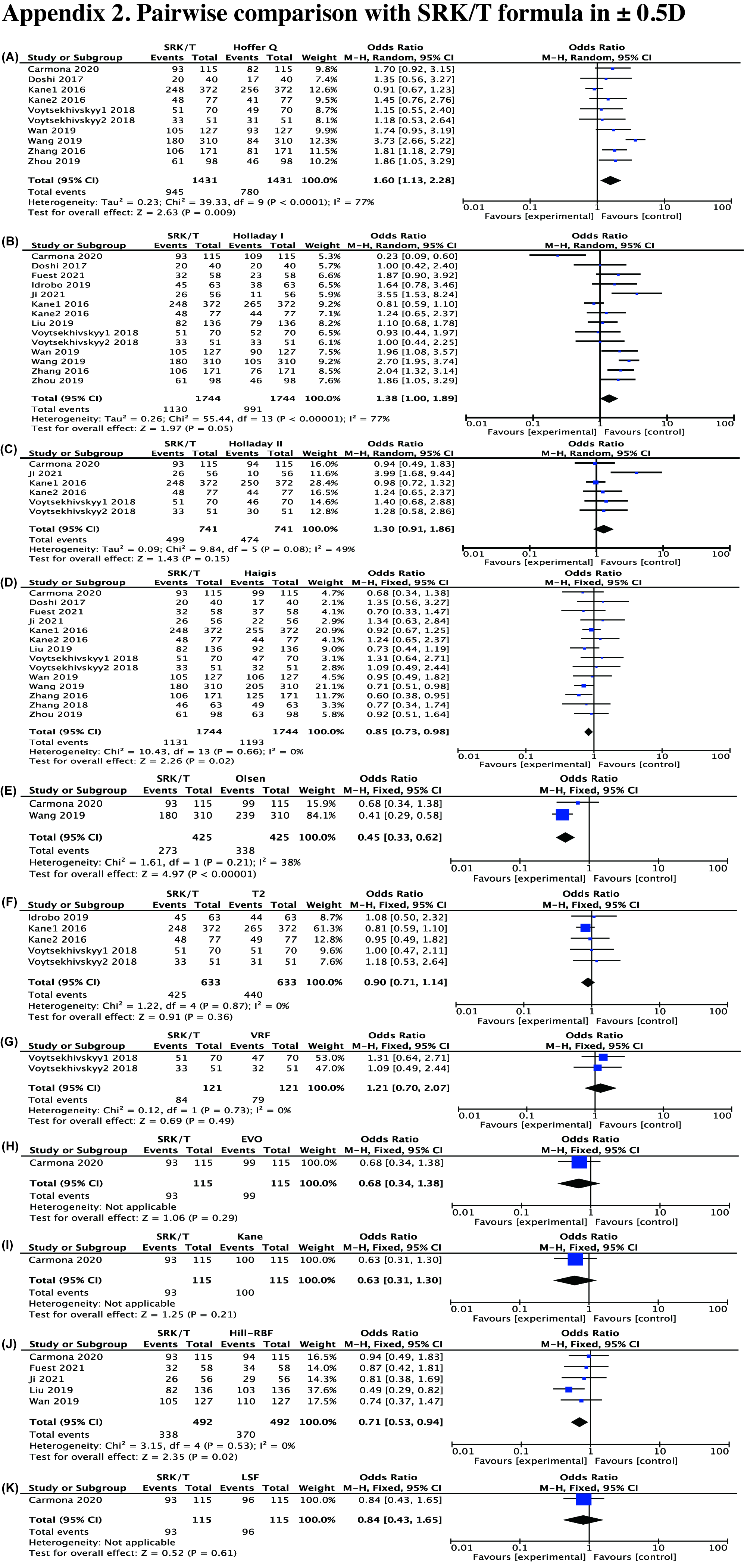

Supplement: Supplementary file 2 — Supplementary file2 (TIF 37288 kb) [file 10792_2022_2466_MOESM2_ESM.tif]

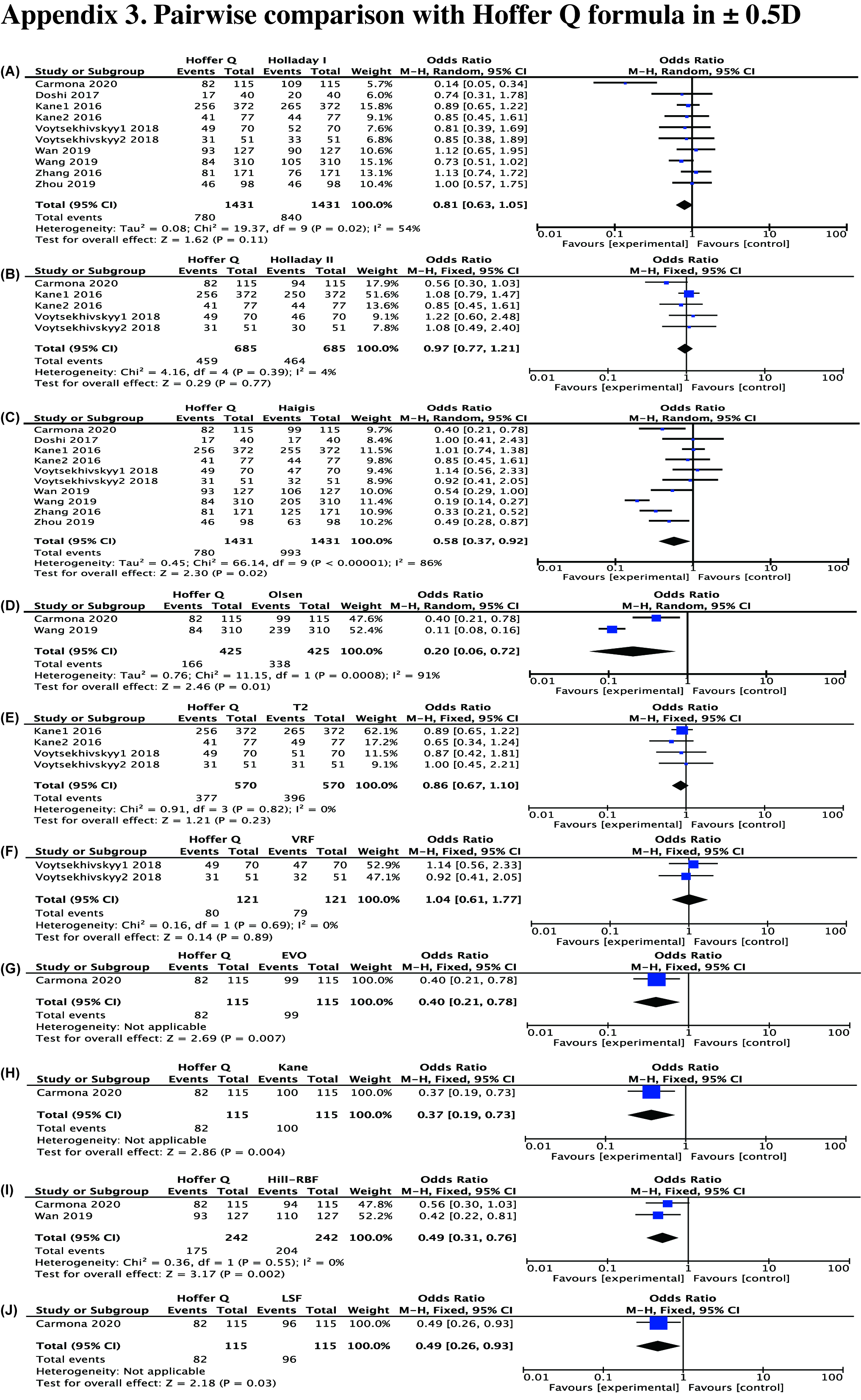

Supplement: Supplementary file 3 — Supplementary file3 (TIF 29959 kb) [file 10792_2022_2466_MOESM3_ESM.tif]

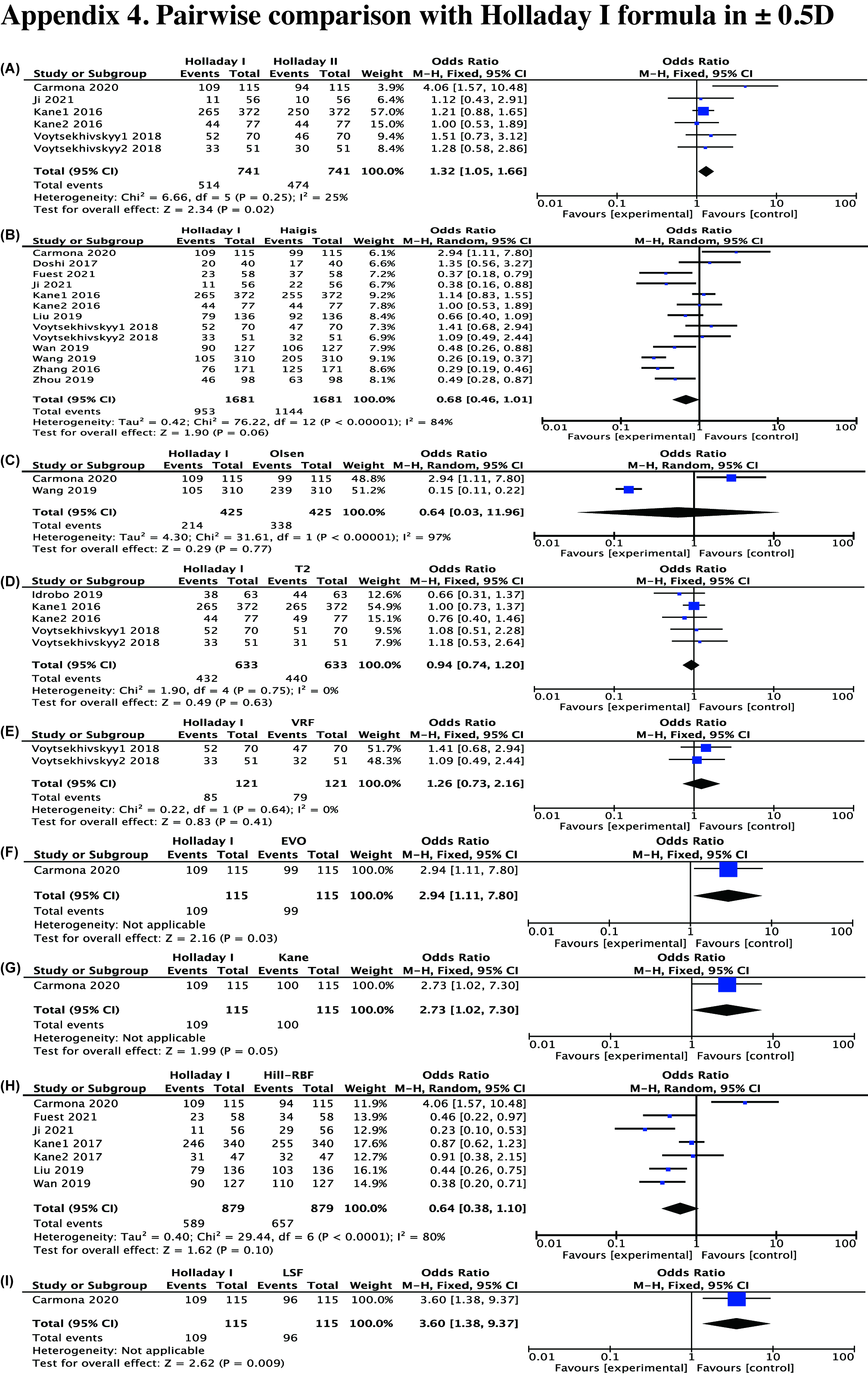

Supplement: Supplementary file 4 — Supplementary file4 (TIF 28856 kb) [file 10792_2022_2466_MOESM4_ESM.tif]

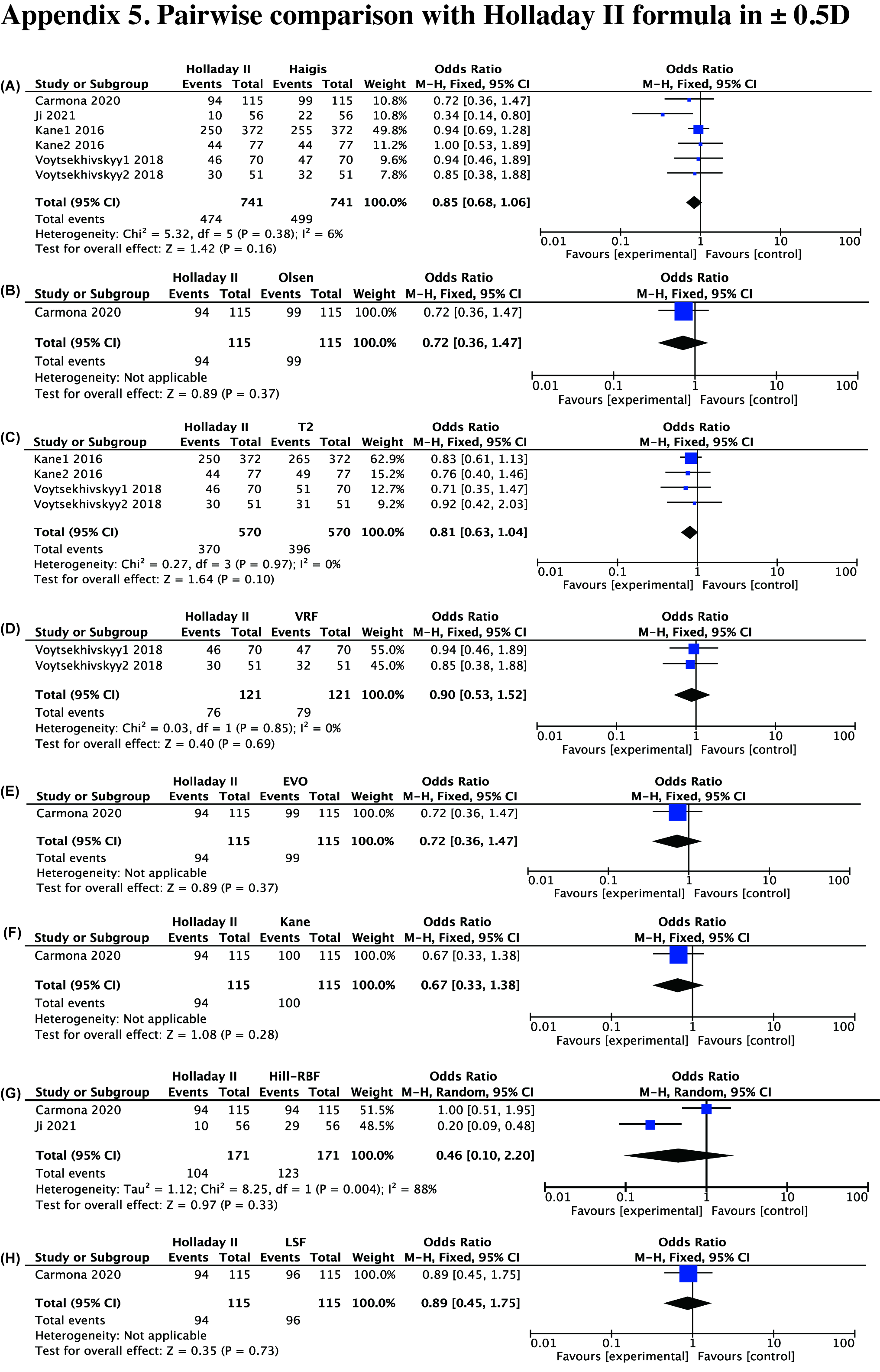

Supplement: Supplementary file 5 — Supplementary file5 (TIF 28486 kb) [file 10792_2022_2466_MOESM5_ESM.tif]

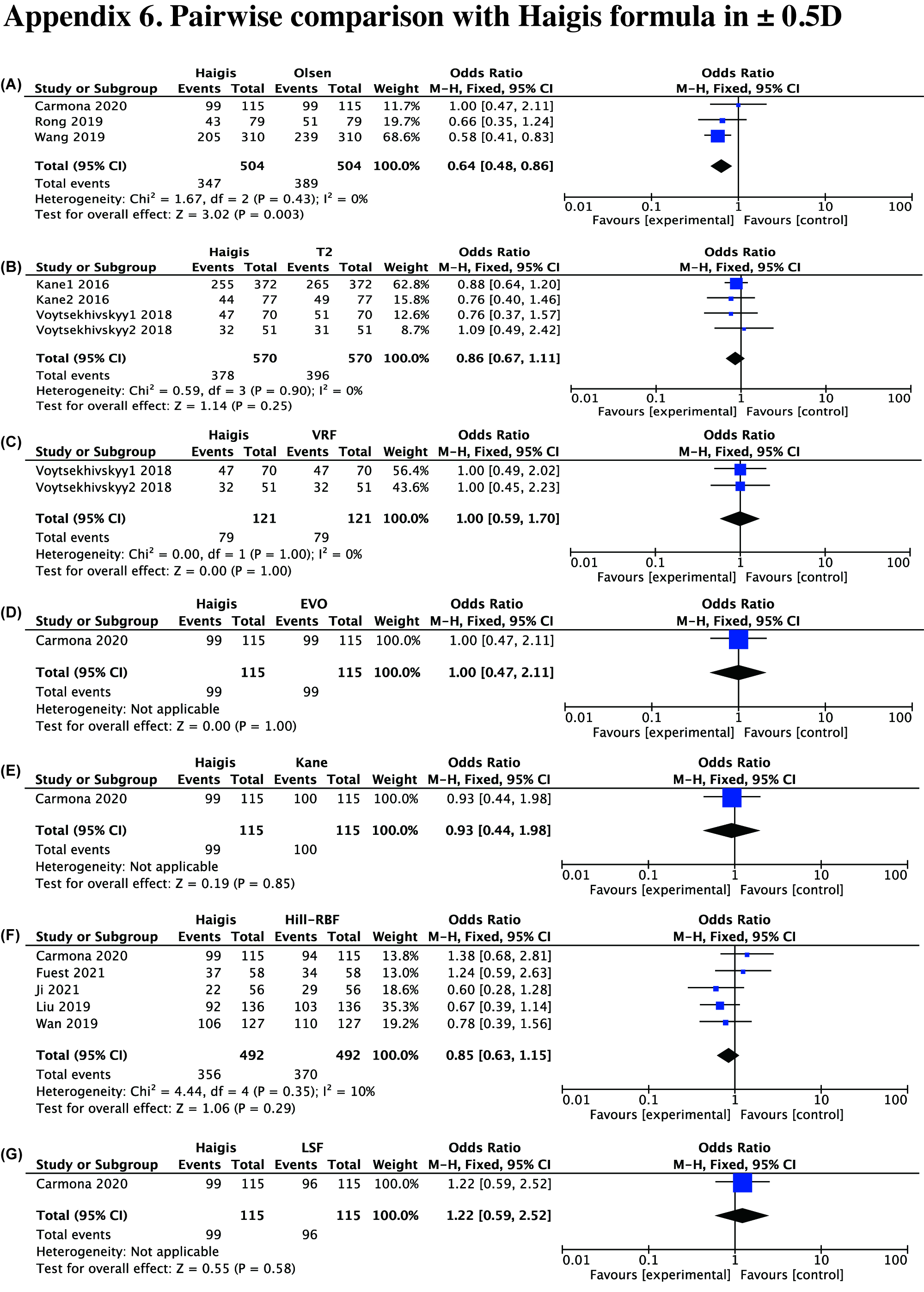

Supplement: Supplementary file 6 — Supplementary file6 (TIF 24823 kb) [file 10792_2022_2466_MOESM6_ESM.tif]

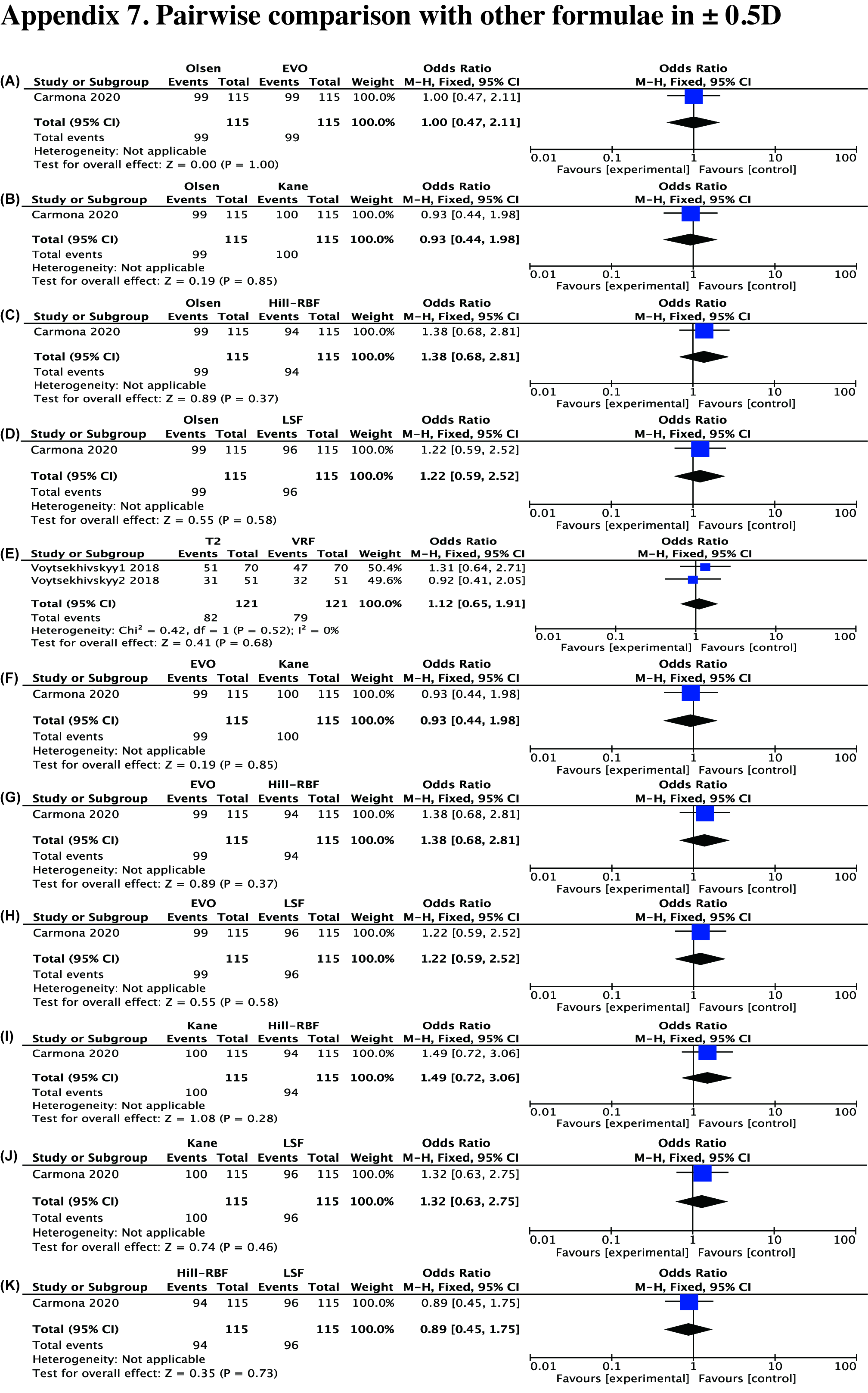

Supplement: Supplementary file 7 — Supplementary file7 (TIF 28927 kb) [file 10792_2022_2466_MOESM7_ESM.tif]

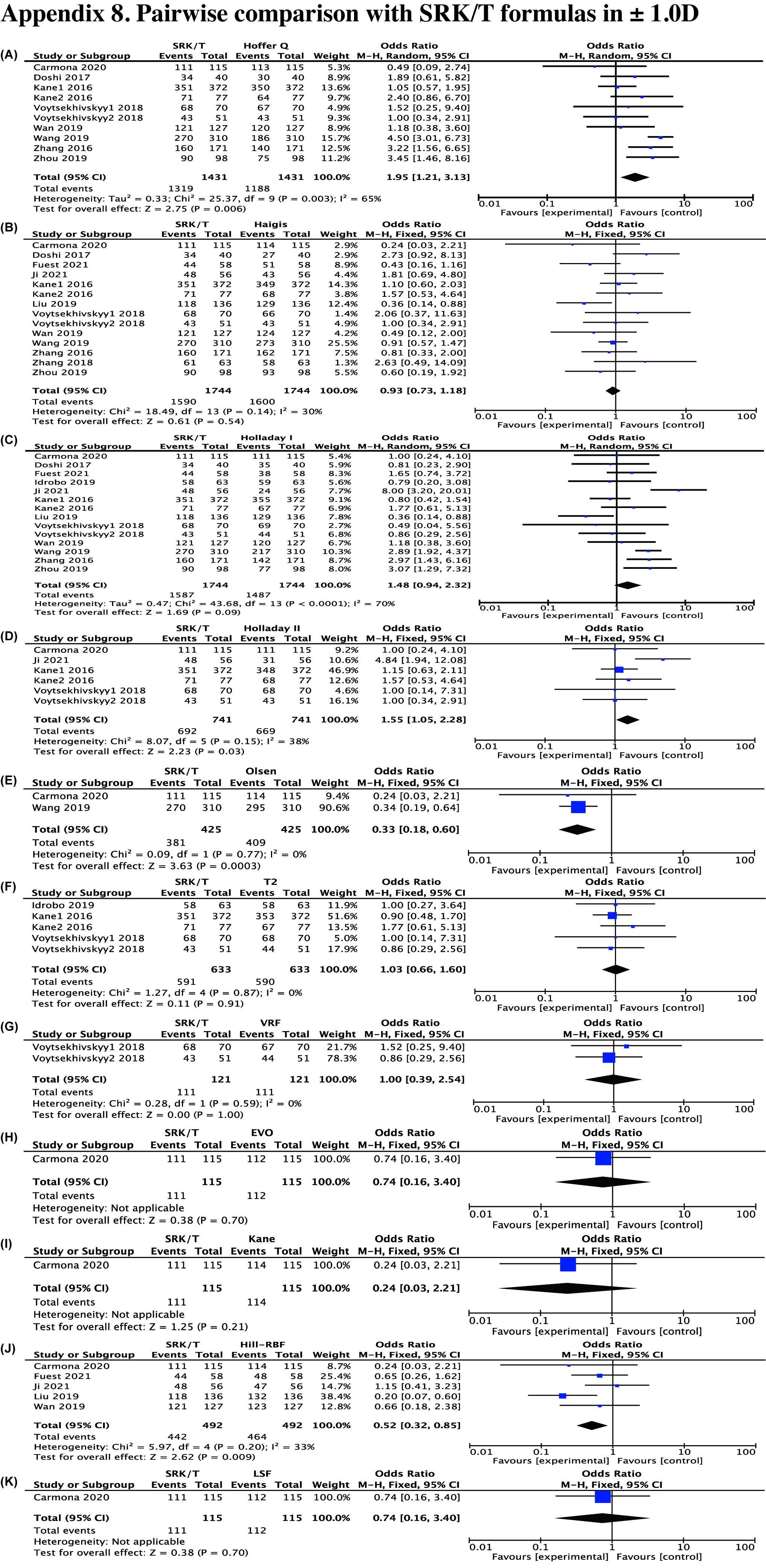

Supplement: Supplementary file 8 — Supplementary file8 (TIF 37811 kb) [file 10792_2022_2466_MOESM8_ESM.tif]

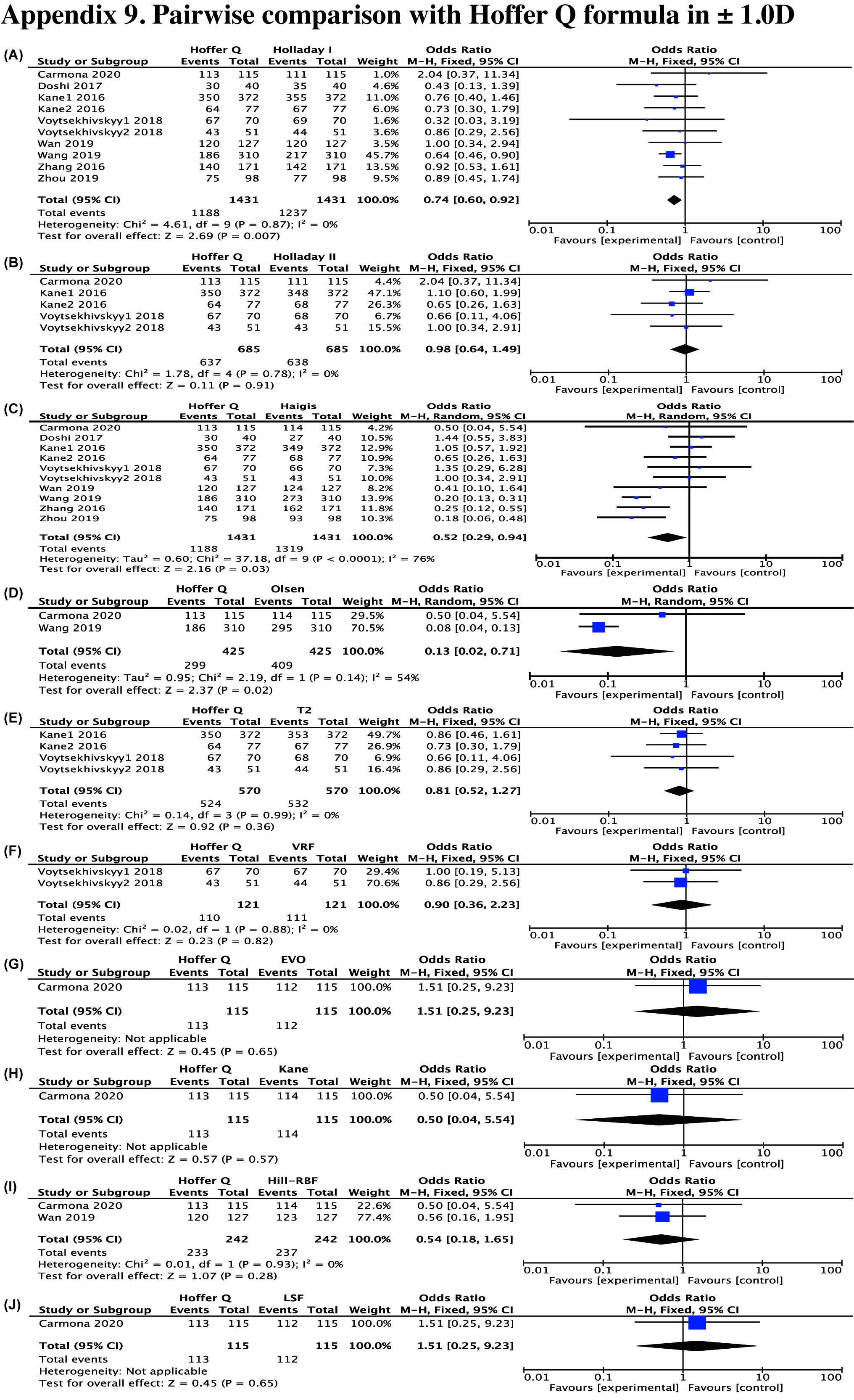

Supplement: Supplementary file 9 — Supplementary file9 (TIF 30212 kb) [file 10792_2022_2466_MOESM9_ESM.tif]

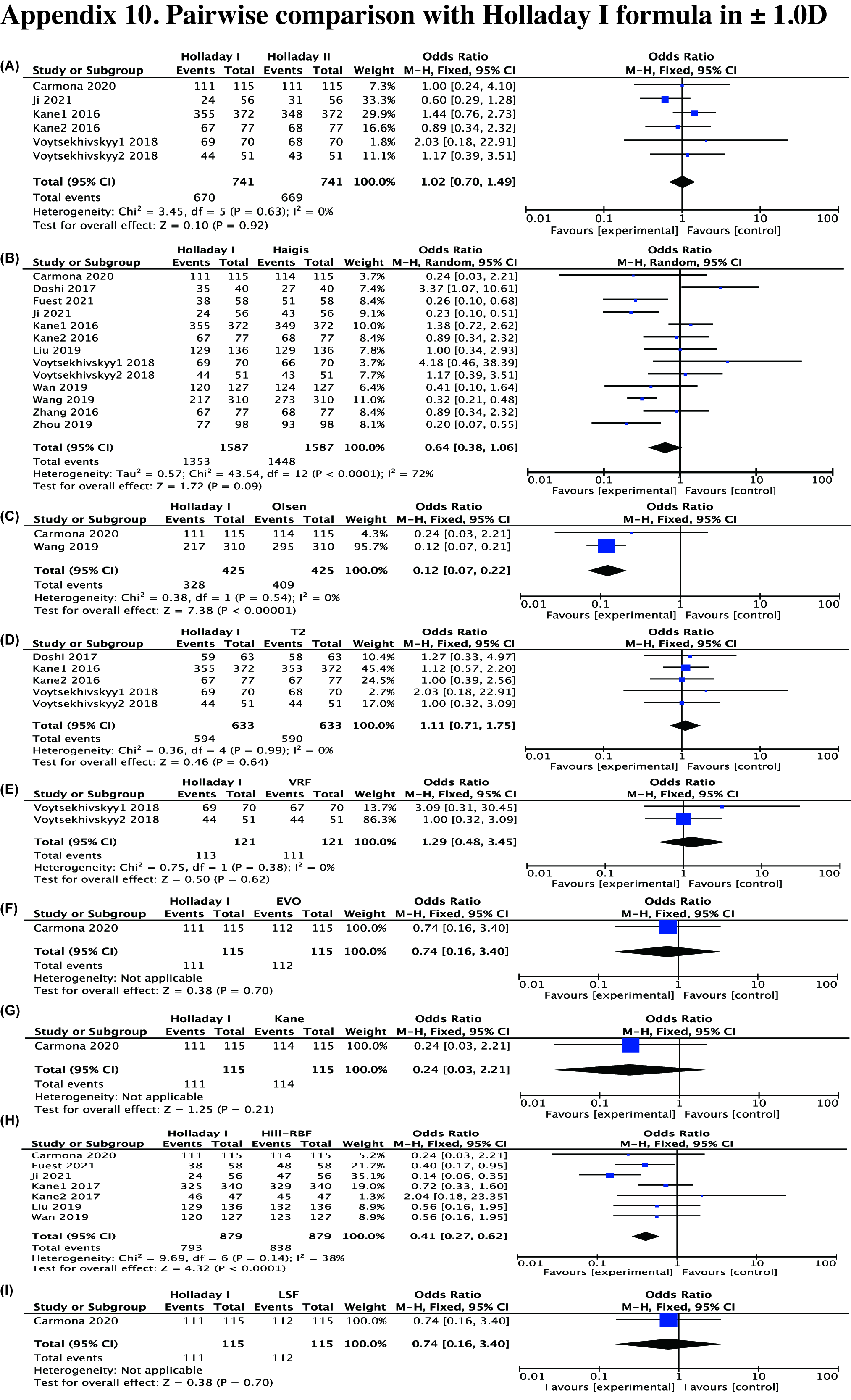

Supplement: Supplementary file 10 — Supplementary file10 (TIF 30066 kb) [file 10792_2022_2466_MOESM10_ESM.tif]

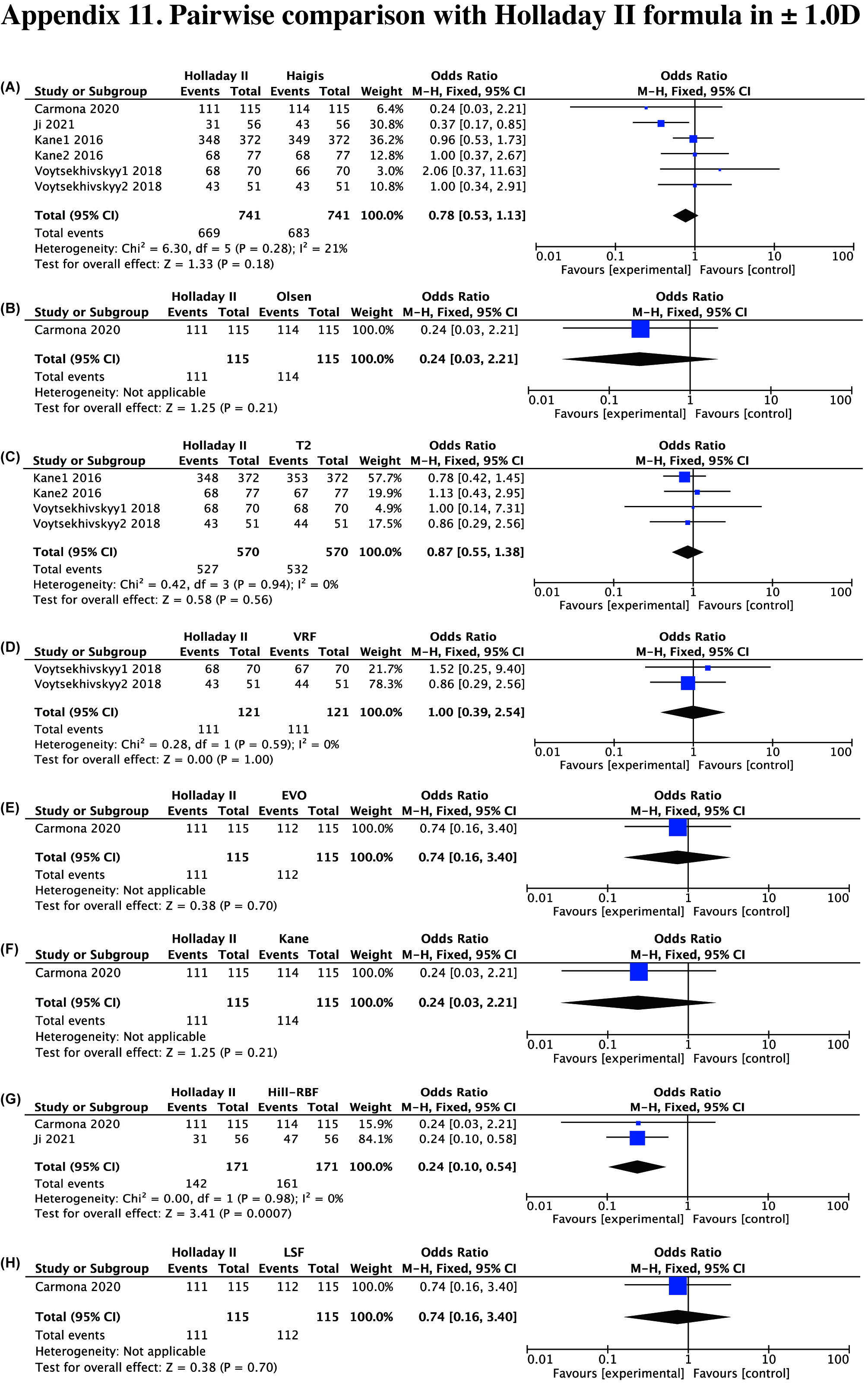

Supplement: Supplementary file 11 — Supplementary file11 (TIF 28607 kb) [file 10792_2022_2466_MOESM11_ESM.tif]

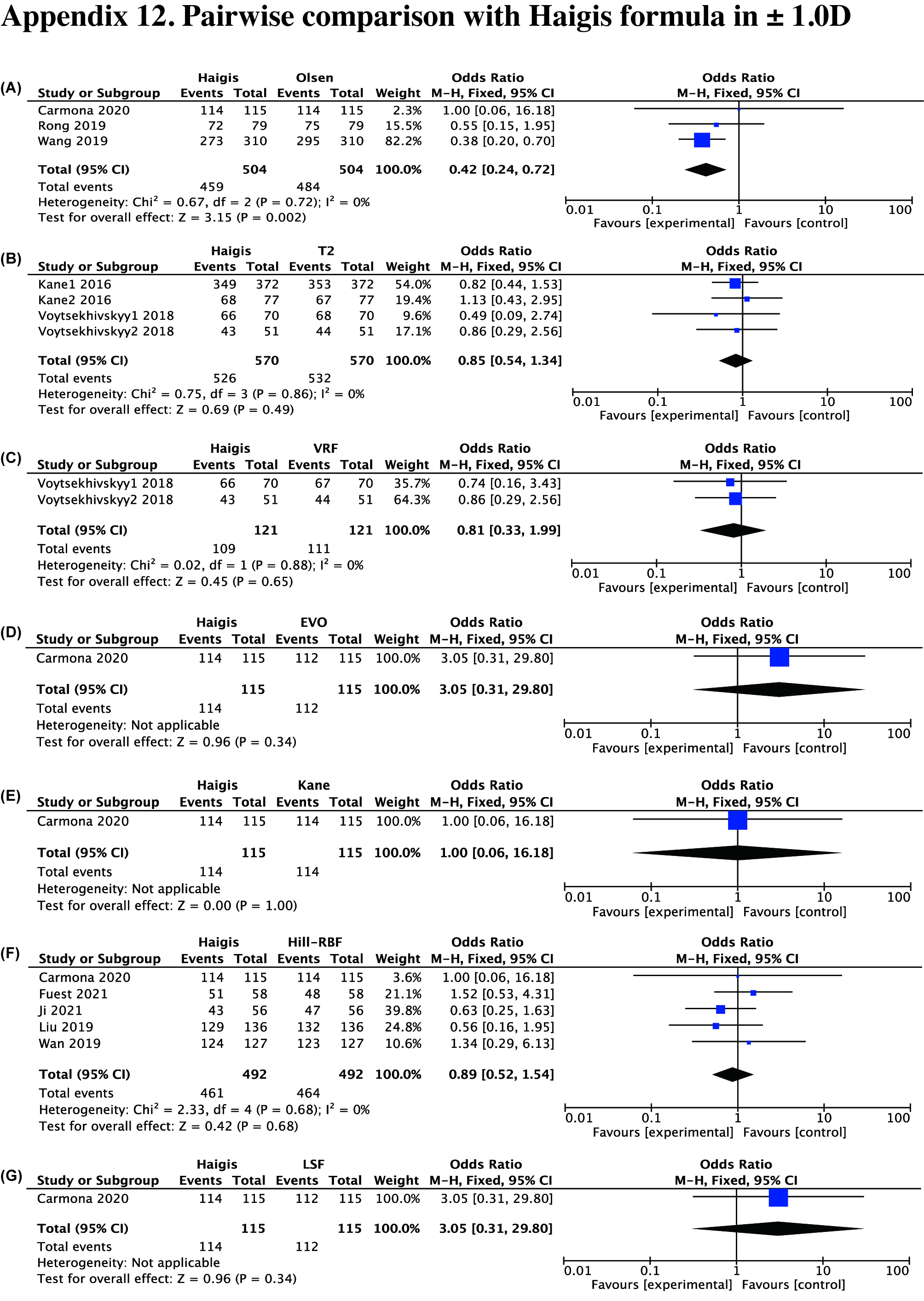

Supplement: Supplementary file 12 — Supplementary file12 (TIF 25181 kb) [file 10792_2022_2466_MOESM12_ESM.tif]

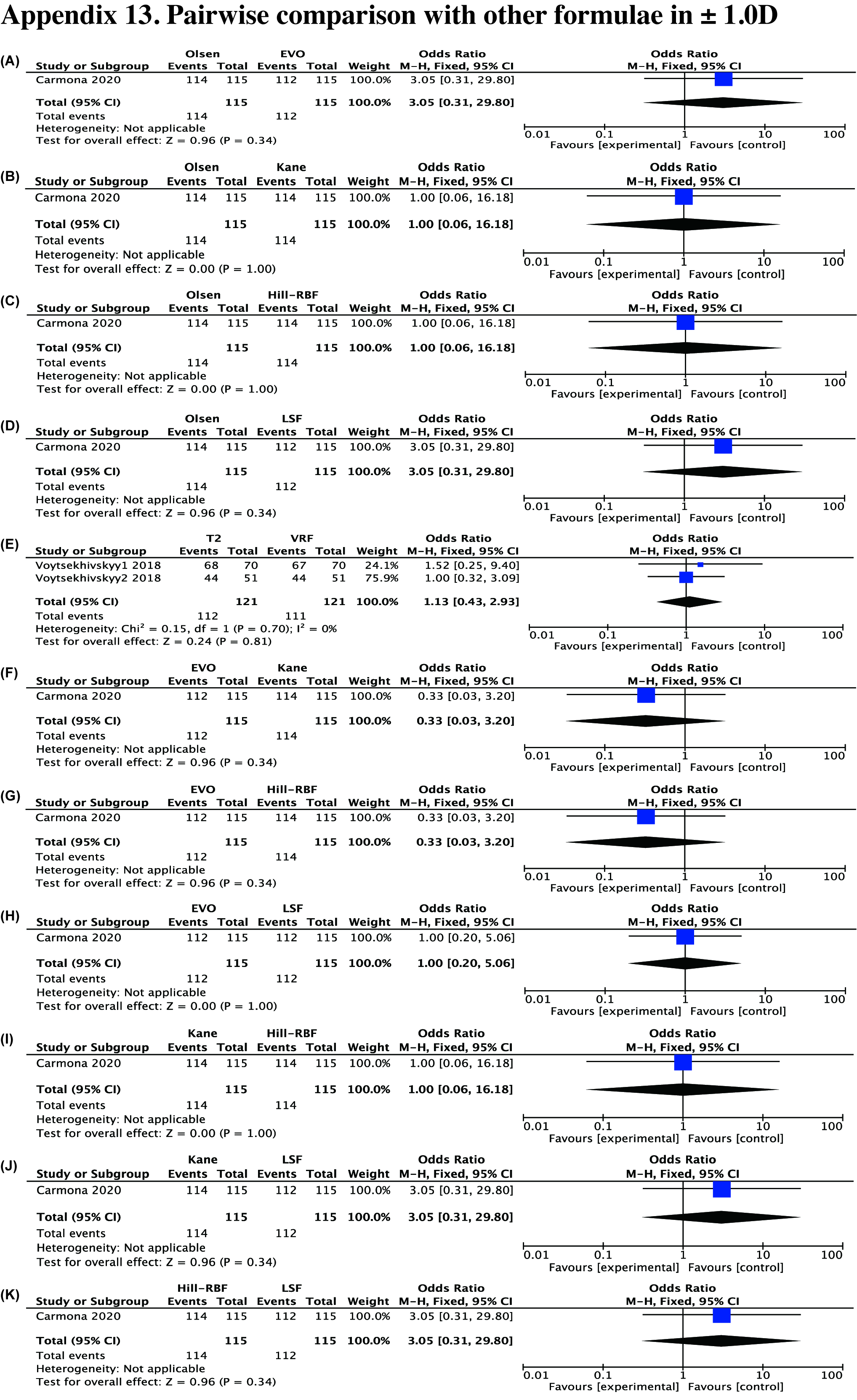

Supplement: Supplementary file 13 — Supplementary file13 (TIF 29976 kb) [file 10792_2022_2466_MOESM13_ESM.tif]

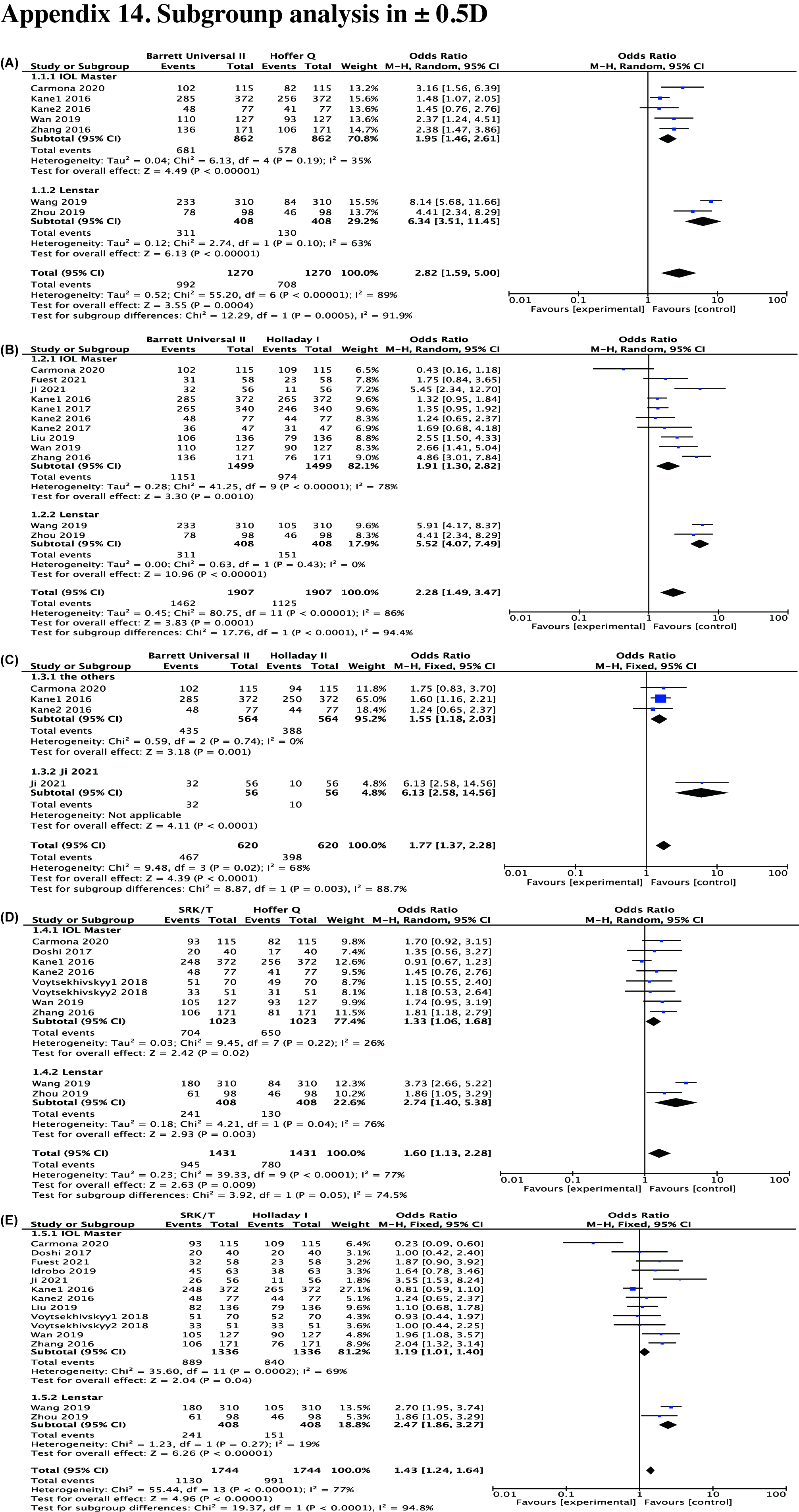

Supplement: Supplementary file 14 — Supplementary file14 (TIF 33923 kb) [file 10792_2022_2466_MOESM14_ESM.tif]

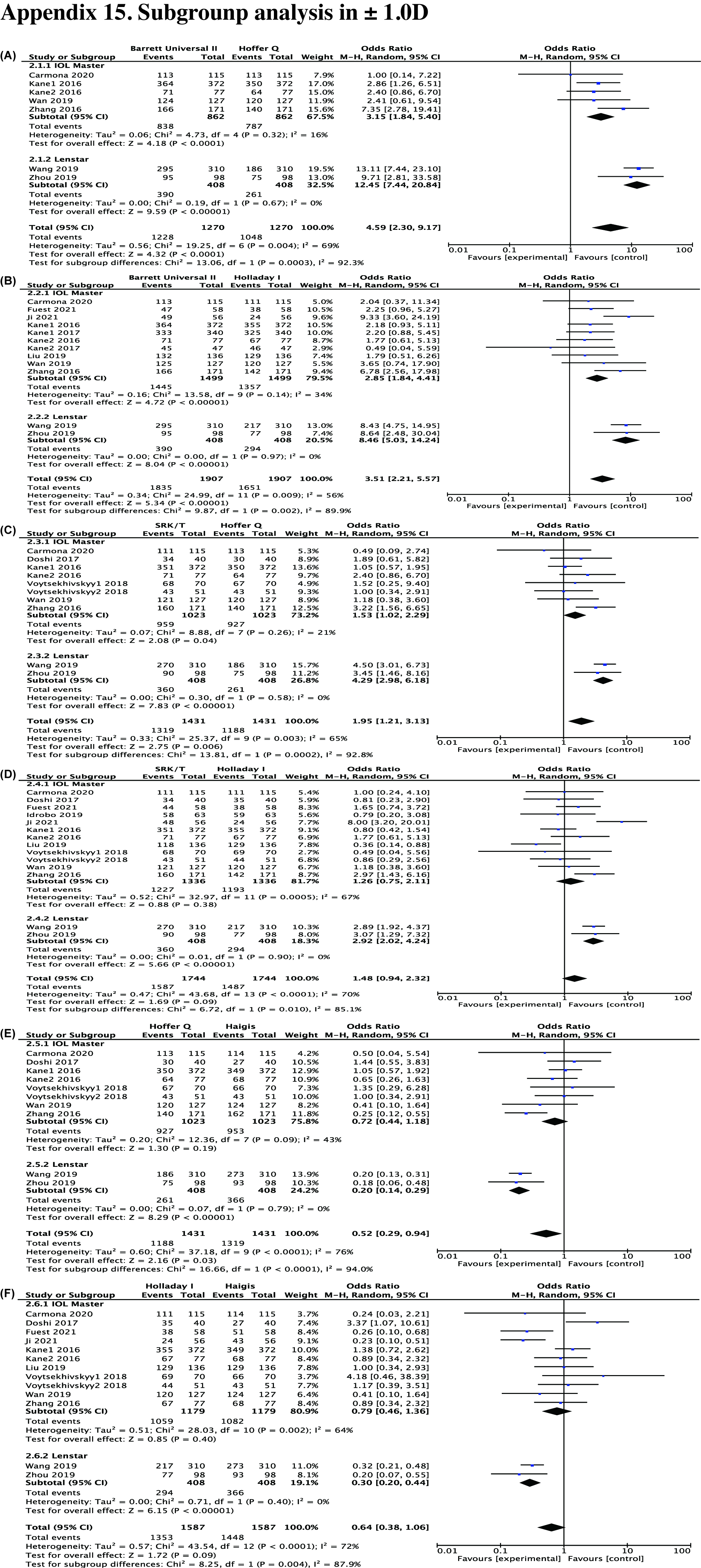

Supplement: Supplementary file 15 — Supplementary file15 (TIF 40038 kb) [file 10792_2022_2466_MOESM15_ESM.tif]

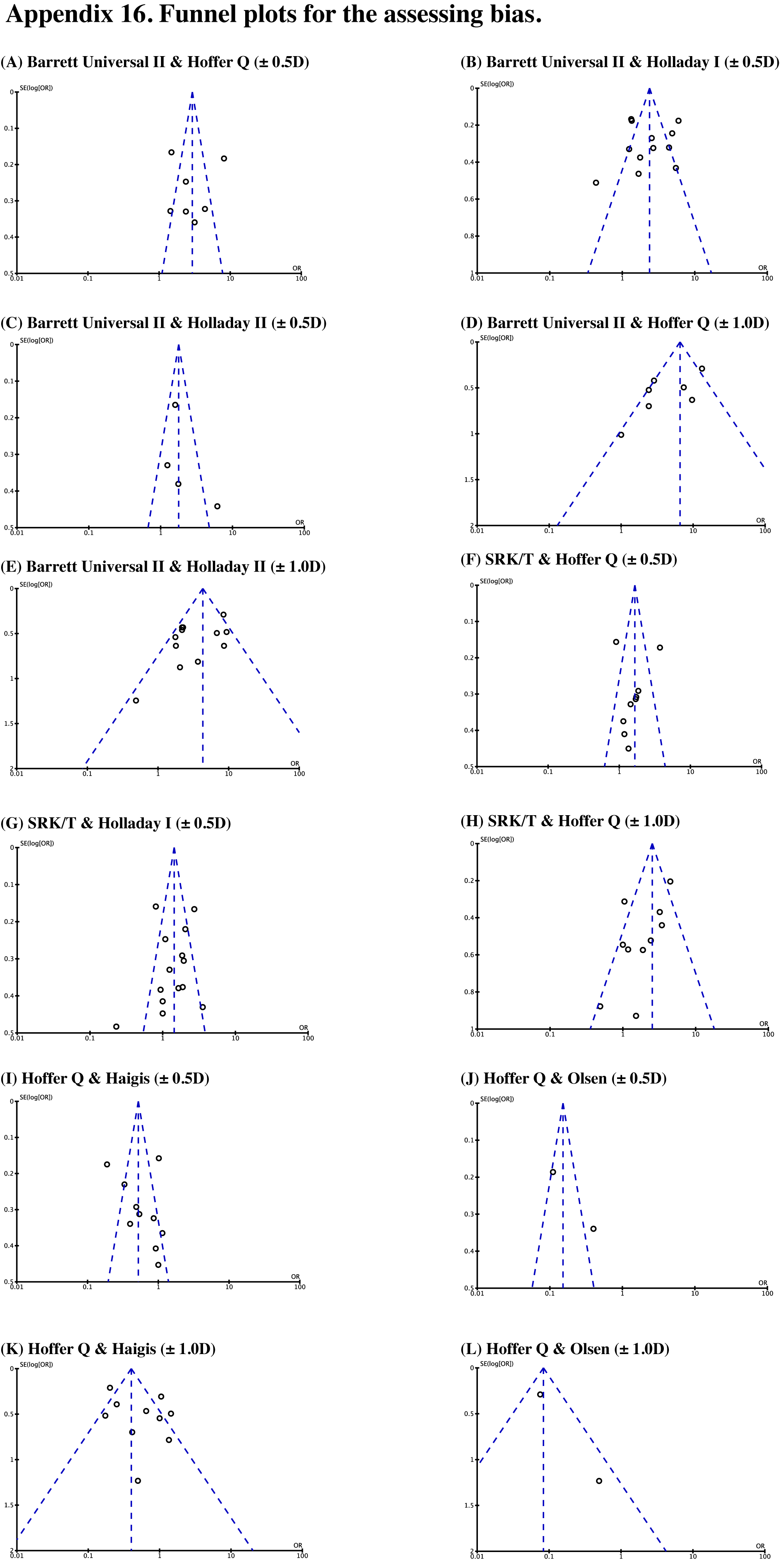

Supplement: Supplementary file 16 — Supplementary file16 (TIF 25037 kb) [file 10792_2022_2466_MOESM16_ESM.tif]
