# Supplementary material for: Comparing the accuracy of the new-generation intraocular lens power calculation formulae in axial myopic eyes: a meta-analysis
Source: Int Ophthalmol. 2022 Sep 5;43(2):619–33. doi: 10.1007/s10792-022-02466-4 (PMC9971158; doi:10.1007/s10792-022-02466-4)
Supplement: Supplementary file 17 — Supplementary file17 (DOCX 21 kb) [file 10792_2022_2466_MOESM17_ESM.docx]

**Methods**

There were two independent investigators (H.Y.L. and Y.L.) searched the databases of PubMed, EMBASE, Web of science and Cochrane library from April 11th to 13th 2021. We searched and selected relevant studies published between Apr 11th, 2011 and Apr 11th, 2021, with using the following terms:

**For PubMed** (available at: <https://www.ncbi.nlm.nih.gov/>)

1# ("cohort studies"[MeSH Terms] OR "case-control studies"[MeSH Terms] OR "comparative study"[Publication Type] OR "risk factors"[MeSH Terms] OR "cohort"[Text Word] OR "compared"[Text Word] OR "groups"[Text Word] OR "case control"[Text Word] OR "multivariate"[Text Word])

2# ("lenses, intraocular"[MeSH Terms] OR "intraocular lenses"[Title/Abstract] OR "lens intraocular"[Title/Abstract] OR "intraocular lens"[Title/Abstract] OR "IOL"[Title/Abstract] OR "IOLs"[Title/Abstract])

#3 ("calculat*"[Title/Abstract] OR "formula*"[Title/Abstract])

#4 ("myopi*"[Title/Abstract] OR "long eye"[Title/Abstract] OR "long axial length"[Title/Abstract] OR "long eyes"[Title/Abstract] OR "long AL"[Title/Abstract] OR "long ALs"[Title/Abstract])

#5 ("last 10 years"[PDat])

#1 AND #2 AND #3 AND #4 AND #5

**For EMBASS** (available at: <https://embase.com/> )

#1 'intraocular lens':ab,ti OR 'intraocular lenses':ab,ti OR iol:ab,ti OR iols:ab,ti OR 'lenses, intraocular':ab,ti OR 'lens intraocular':ab,ti

#2 calculat*:ab,ti OR formula*:ab,ti

#3 'clinical article'/exp OR 'controlled study'/exp OR 'major clinical study'/exp OR 'prospective study'/exp OR 'cohort analysis'/exp OR 'cohort':ti,ab OR 'compared':ti,ab OR 'groups':ti,ab OR 'case control':ti,ab OR 'multivariate':ti,ab

#4 myopi*:ab,ti OR 'long eye':ab,ti OR 'long eyes':ab,ti OR 'long al':ab,ti OR 'long als':ab,ti OR 'long axial length':ab,ti OR 'long axial lengths':ab,ti

#1 AND #2 AND #3 AND #4

**For Web of Science** (available at: <http://apps.webofknowledge.com/> )

#1AB=(intraocular lens) OR AB=( intraocular lenses) OR AB=(lens, intraocular) OR AB=(IOL) OR AB=(IOLs)

#2 AB=(calculat*) OR AB=(formula*)

#3AB=(myopi*) OR AB=(long eye) OR AB=(long eyes) OR AB=(long axial length) OR AB=(long axial lengths) OR AB=(long AL) OR AB=(long ALs)

#4 #1 AND #2 AND #3 time span =2011-2021 search language=automatic

**For Cochrane library** (available at: <https://www.cochranelibrary.com>)

#1 MeSH descriptor: [Lenses, Intraocular] explode all trees

#2 (intraocular lenses):ti,ab,kw

#3 (lens intraocular):ti,ab,kw

#4 (IOL*):ti,ab,kw

#5 #1 OR #2 OR #3 OR #4

#6 (myopi*):ti,ab,kw

#7 (long axial length*):ti,ab,kw

#8 (long eyes*):ti,ab,kw

#9 (long AL*):ti,ab,kw

#10 #6 OR #7 OR #8 OR #9

#11 (calculat*):ti,ab,kw

#12 (formula*):ti,ab,kw

#13 #11 OR #12

#14 #5 AND #10 AND #13 with Publication Year from 2011 to 2021, in Trials
